# Structural and Photophysical Modulation of Au(I)–Pb(II) Chains by Solvent Inclusion: A Study of Solvatopolymorphs

**DOI:** 10.1021/acs.organomet.5c00275

**Published:** 2025-09-25

**Authors:** David Royo, Sonia Moreno, María Rodríguez-Castillo, Miguel Monge, M. Elena Olmos, Fedor Zubkov, Anastasia A. Pronina, Ghodrat Mahmoudi, José M. López-de-Luzuriaga

**Affiliations:** † Departamento de Química, Instituto de Investigación en Química (IQUR), Complejo Científico Tecnológico, Universidad de La Rioja, Madre de Dios 53, 26006 Logroño, Spain; ‡ Department of Organic Chemistry, RUDN University, 6 Miklukho-Maklaya St, Moscow 117198, Russia; § Department of Chemistry, Faculty of Science, University of Maragheh, P.O. Box 55136-83111, Maragheh 97HF+498, Iran; ∥ Samara State Technical University, Molodogvardeyskaya Str 244, Samara 443100, Russia

## Abstract

This work presents the synthesis, structural characterization, and photophysical studies of a new gold­(I)–lead­(II) heterometallic polymeric complex, [{Au­(C_6_F_5_)_2_}_2_{Pb­(S-Terpy)}]_*n*_, and its solvatopolymorphs incorporating acetone and diethyl ether. The complex exhibits reversible solvatochromic behavior with significant changes in color and luminescence upon solvent inclusion. X-ray diffraction reveals that the trinuclear Au–Pb–Au units are maintained across all solvatopolymorphs, although the packing and intermetallic distances vary depending on the solvent and its coordination mode. Optical studies show that the complexes emit in the visible to near-infrared region, with emission properties strongly influenced by the nature of the solvent. DFT and TD-DFT reveal that the emission arises from metal-to-ligand or ligand-to-ligand charge transfer transitions, depending on the structure. These results highlight the potential of gold–lead supramolecular systems as responsive luminescent materials for sensing applications.

## Graphical abstract

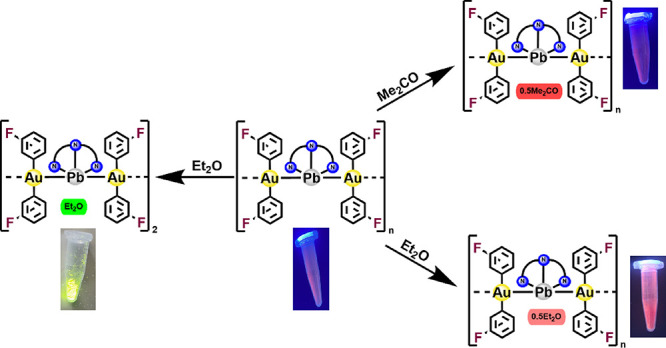

## Introduction

During the last years, scientists have paid special attention to noncovalent interactions, both from a theoretical and from an experimental point of view, mainly due to the effect that they may have on the properties of the systems in which they are present. Among them, intermolecular interactions have been shown to be the origin of the formation of supramolecular materials that may display multiple astonishing structures with diverse and interesting properties. Perhaps, the most frequent in gold complexes, together with hydrogen bonding, are aurophilic interactions,^1^ which, in the case of heterometallic compounds containing other closed-shell metal ions, often appear accompanied by metallophilic interactions.^2^ These two types of intermetallic interactions have received increasing interest because they are associated with intriguing physical and chemical properties,^3^ such as luminescence. The strength and directionality of these interactions, as well as the number and type of ligands bonded to the metal atoms, influence the optical properties observed. For instance, an unprecedented and unequivocal relationship between the emission energy and the Au–Au distance in a gold/silver complex has recently been described.^4^

Besides, a field that attracts growing attention is the study of the changes that various external stimuli (such as pressure,^5^ temperature,^6^ vapors,^6b,6c^ or solvents)^6a,7^ may provoke in the luminescence of homo- or heterometallic gold systems,^8^ which is of undoubtedly great interest for their potential application as sensors.^9^ Obviously, such significant changes in color and/or luminescence upon exposure to these external stimuli are related to the structural modifications that they induce in these compounds, which leads us to introduce the concept of polymorphism. This concept was defined by McCrone as “a solid crystalline phase of a given compound resulting from the possibility of at least two different arrangements of the molecules of that compound in the solid state”.^10^

A special type of polymorphism is solvatomorphism, which appears in “systems where the crystal structures of the substance are defined by different unit cells, but where these unit cells differ in their elemental composition through the inclusion of one or more molecules of solvent”.^11^ Introducing these molecules into the network, as solvation molecules in solution or as vapors, frequently causes significant changes in the crystal packing, even if there is no interaction between the solvent and the metal centers. These modifications alter the interactions within the crystal, leading to noticeable color changes that are often visible to the naked eye and even more pronounced under UV light. An example of this behavior was recently found in the trinuclear gold­(I) complex [Au_3_(MeIm)_3_], where the inclusion of dichloromethane provokes structural changes as the appearance of hydrogen bonds involving the solvent and the complex, as well as aurophilic interactions, which lead to the formation of a dimer.^12^

If we focus on the above-mentioned luminescent gold-heterometal complexes, there are various materials incorporating different metals, such as copper,^13^ silver,^14^ and lanthanides,^15^ and some even show stimulus-responsive properties.^16^ Our group treasures a vast experience in the synthesis and characterization of such materials, and in a number of examples they have shown mecano-, vapo-, and/or solvatochromic properties. Regarding solvatochromic materials, our contributions include the heterometallic gold­(I)–thallium­(I) polymer {Tl­[Au­(C_6_Cl_5_)_2_]}_*n*_, which is able to react with various organic solvents such as THT, THF, and acetone, exhibiting reversible solvatochromism when heated up to 100 °C.^17^ Another example involves a series of gold­(I)–silver­(I) derivatives based on the tetranuclear unit [Au_2_Ag_2_R_4_] (R = perhalophenyl group) that display either reversible or irreversible behavior depending on the donor capacity of the vapors to silver and the nature of the perhalophenyl rings.^18^

In the case of gold­(I)–lead­(II) compounds, their chemistry has been developed to a much lesser extent since Professor Fackler Jr. published the first complex displaying Au­(I)···Pb­(II) interactions in 1989, although they were supported by the bridging ligand [CH_2_P­(S)­Ph_2_]^−^.^19^ Our research group has significantly contributed to the progress in the area of systems formed via unsupported Au···Pb interactions in the past decade.^20^ In one of our last contributions, we reported the polymeric complex [{Au­(C_6_F_5_)_2_}_2_{Pb­(terpy)}]_*n*_, which displays reversible responses to solvents and vapors when exposed to acetonitrile, toluene, or tetrahydrofuran.^21^ In this case, the organic solvent molecules are accommodated in the holes of the crystal structure, and while the sequence of metals remains unaltered and the changes occur in the coordination environment of lead­(II), this solvation process results in a change in the color and luminescence of the starting compound. Another recent example from our research group deals with the ability of the benzonitrile solvate of the same polymer, {[{Au­(C_6_F_5_)_2_}_2_{Pb­(terpy)}]·NCPh}_*n*_, to suffer an unprecedented reorientation and coordination of the solvent molecules to the lead centers upon exposure to high pressures or by increasing the temperature, effects that have a strong influence on the photophysical properties of these two polymorphs.^22^

To deepen our knowledge of supramolecular systems built via noncovalent interactions, as unsupported Au···Pb ones, in this article, we report a complete study of a gold­(I)–lead­(II) compound containing a terpyridine-type ligand functionalized with a thiofuran ring (S-Terpy), which could, in theory, allow for an increase of the molecular complexity of the new compounds thanks to the donor ability of the free sulfur atom present in the functional group of the ligand. In this paper, we describe the synthesis, structural characterization, and reversible response to solvents, as well as a computational study of the complex [{Au­(C_6_F_5_)_2_}­{Pb­(S-Terpy)}]_*n*_ and its diethyl ether and acetone solvatopolymorphs. It is worth noting that both the nature of the solvent and the metal/solvent ratio highly modify the photophysical properties of the solvatopolymorphs, which surprisingly display unusual emissions in the NIR region.

## Results and Discussion

### Synthesis and Characterization of the Complexes

By reaction of [Au_2_Ag_2_(C_6_F_5_)_4_(OEt_2_)_2_]_*n*_ with PbCl_2_, dissolved in methanol, and the subsequent addition of S-Terpy dissolved in dichloromethane, in a 2:1:1 molar ratio, we obtained polymeric complex [{Au­(C_6_F_5_)_2_}_2_{Pb­(S-Terpy)}]_*n*_ (**1**). This complex is stable to air and moisture for long periods, and its analytical and spectroscopic data are in accordance with the proposed formulation (see Experimental and Electronic Supporting Information).

Interestingly, when complex **1** is treated with, in principle, innocent organic solvents such as acetone or diethyl ether, the new gold­(I)–lead­(II) heterometallic compounds {[{Au­(C_6_F_5_)_2_}_2_{Pb­(S-Terpy)}]·0.5Me_2_CO}_*n*_ (**2**), {[{Au­(C_6_F_5_)_2_}_2_{Pb­(S-Terpy)}]·0.5Et_2_O}_*n*_ (**3**), and {[{Au­(C_6_F_5_)_2_}_2_{Pb­(S-Terpy)}]·Et_2_O}_2_ (**4**) and) are obtained (see Scheme 1). These complexes, as we will see, incorporate the new solvent molecules in their structures, without interacting with the metal centers, maintaining the same metal sequence within the trimetallic unit, Au···Pb···Au, but differing in distances and packing arrangements and, as will be commented below, in their optical properties.

**Scheme 1:**
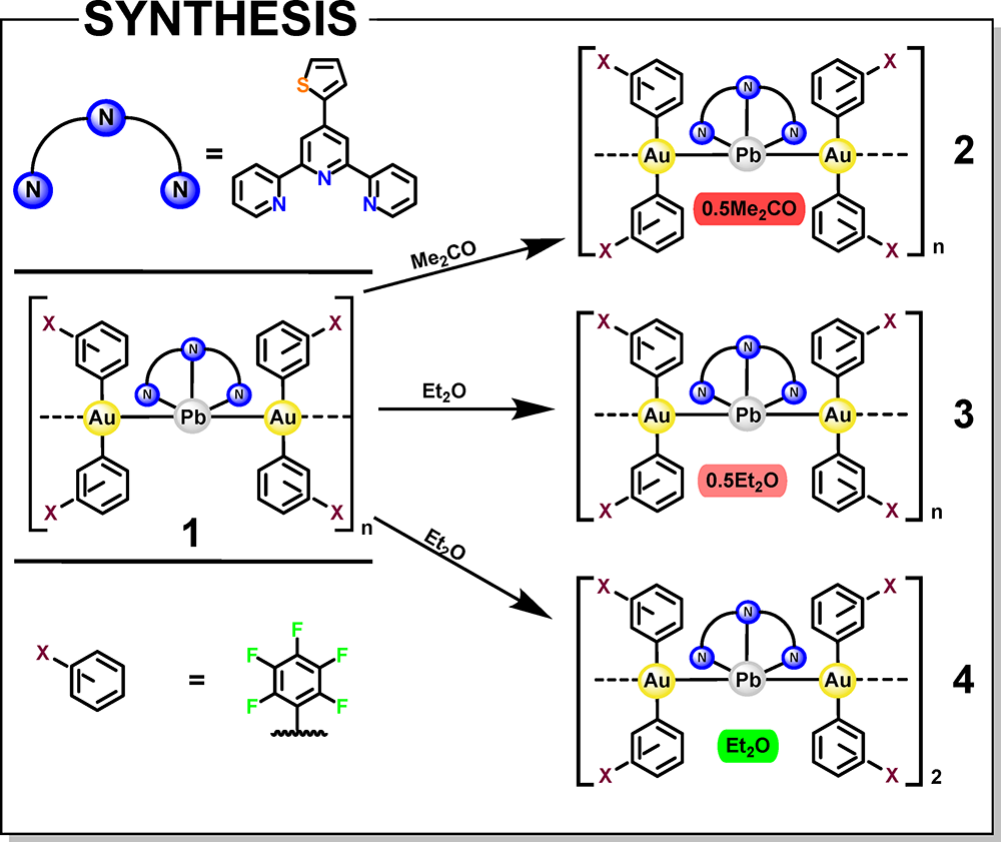
Synthesis of Complexes **1–4**

The IR spectra of the new four complexes are almost identical; they all display the expected vibrations due to the C=N and C=C bonds of the S-Terpy ligand around 1600 and 1450 cm^–1^, respectively, and the absorptions due to the pentafluorophenyl groups bonded to gold­(I) at about 1500, 950, and 785 cm^–1^. In the case of solvatopolymorph **2**, an additional band at 1700 cm^–1^, which is associated with the stretching mode of the C=O bond of the acetone molecules, also appears. In the cases of **3** or **4**, the bands associated with the solvent, diethyl ether, appear at 1051 or 1054 cm^–1^, respectively, which correspond to the stretching mode of the C–O bond of this molecule (see the ESI).

Regarding their ^1^H NMR spectra in [D_6_]-DMSO, all complexes show eight signals corresponding to the S-Terpy ligand in the range 8.79–7.28 ppm, which are shifted from those of the free ligand, indicating its coordination to lead in solution. In addition, the signals due to the solvent molecules in solvatopolymorphs **2**, **3,** and **4** are also observed. Thus, in complex **2**, the methyl groups of the acetone molecules appear as a singlet at 2.05 ppm, while those of the methyl protons of the diethyl ether molecules appear as triplets at 0.92 (**3**) and 1.07 ppm (**4**), respectively. This difference in the diethyl ether signals could be attributed to the fact that, at least in the solid state, some hydrogen atoms of the methyl groups in compound **4** engage in hydrogen bonding with fluorine, whereas in compound **3**, these hydrogens do not participate in such interactions. If these hydrogen bonds in **4** were maintained in solution, this would account for the greater deshielding of the ether methyl protons in compound **4** (1.07 ppm) compared to compound **3** (0.92 ppm). Nevertheless, the resonances corresponding to the methylene groups in **3** and **4**, which should appear at about 3.4 ppm, are masked by those of the water present in the deuterated solvent.

On the other hand, the ^19^F NMR spectra of the four complexes display the signals corresponding to the inequivalent fluorine atoms of the C_6_F_5_ groups bonded to gold­(I) at similar chemical shifts: −114.5 (m, 4F, F_o_), −161.50 (t, 2F, F_p_, ^3^ *J*(F_p_–F_m_) ≈ 20 Hz), and −162.8 (m, 4F, F_m_) ppm. This coincidence in shifts is in accordance with the conductivity measurements for the four complexes that are typical of 1:2 electrolytes and therefore with the dissociation of the cationic and anionic counterparts for the four complexes in solution (see the Experimental Section).

Finally, the MALDI(−) mass spectra of the four complexes display in all cases the peak corresponding to the [Au­(C_6_F_5_)_2_]^−^ fragment at *m*/*z* = 531 as the base peak, while in their MALDI­(+) mass spectra the base peak corresponds to the fragment [{Au­(C_6_F_5_)_2_}­{Pb­(S-Terpy)}]^+^ at *m*/*z* = 1054, which corroborates the formation of supramolecular entities in solid state, presumably via intermetallic Au···Pb interactions.

### X-ray Crystal Structure Determination

The crystal structures of complexes **2**, **3**, and **4** were determined by single-crystal X-ray diffraction on crystals grown via slow diffusion of *n*-hexane into saturated solutions of the complexes in acetone (**2**) or diethyl ether (**3** and **4**).

As shown in Figure 1, the crystal structures of the three solvatopolymorphs contain common building blocks, trinuclear Au_2_Pb units [{Au­(C_6_F_5_)_2_}­{Pb­(S-Terpy)}­{Au­(C_6_F_5_)_2_}], within which the metal atoms are joined through two unsupported Au···Pb interactions. In the case of complex **3**, the asymmetric unit contains two trinuclear Au_2_Pb fragments, although in Figure 1 only one of them is represented. The difference between these building blocks is the nature or the amount of solvent present in each case. Thus, while **2** contains half a molecule of acetone per Au_2_Pb fragment, **3** and **4** crystallize with diethyl ether, although in different Au_2_Pb:solvent ratio, 1:0.5 or 1:1, respectively. It is worth noting that only in the case of complex **2** the solvent interacts with the lead center through its oxygen atom, while in the crystal structures of **3** and **4** the diethyl ether is connected to the trimetallic unit through C–H···O and C–H···F hydrogen bonds. Besides, the [Au­(C_6_F_5_)_2_]^−^ anions and the central [Pb­(S-Terpy)]^2+^ cation within the trinuclear unit are also somehow held together by π–π stacking interactions.

**Figure 1:**
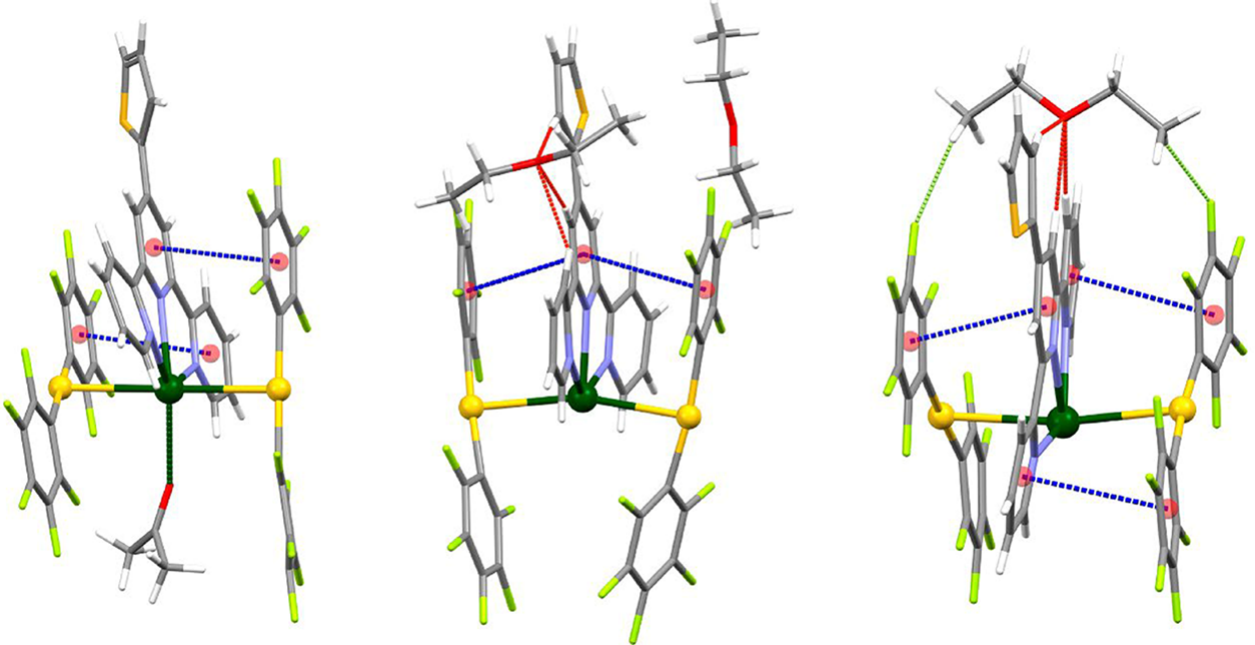
Trinuclear Au_2_Pb building blocks in the crystal structures of complexes **2** (left), **3** (center), and **4** (right). Color code: gold (yellow), lead (green), oxygen (red), nitrogen (purple), sulfur (orange), carbon (gray), fluorine (light green), π–π interactions (dashed blue), C–H···O (dashed red), and C–H···F (dashed purple) hydrogen bonds.

The Au–Pb distances are very similar in the structures of **2** [2.9776(6) and 2.9578(7) Å] and **4** [2.9661(10) and 2.9645(10) Å], while complex **3** displays slightly shorter Au–Pb distances [from 2.917(4) to 2.856(4) Å]. Nevertheless, all of them compare well with those described for the related vapochromic complex [{Au­(C_6_F_5_)_2_}_2_{Pb­(terpy)}]_*n*_ and its acetonitrile, toluene and tetrahydrofuran polymorphs, were the Au–Pb distances range from 2.9740(5) to 2.8349(4) Å [17, 18], as well as with the Au–Pb distances observed in the Au–Pb–Au fragments of the decanuclear Au_6_Pb_4_ complex [{Au­(C_6_F_5_)_2_}_6_{PbCl­(terpy)}_2_{Pb­(terpy)}_2_] (2.9332(9)-2.8776(9) Å) [16a] and with those found in [Au_2_Pb­(CH_2_P­(S)­Ph_2_)_4_] (2.963(2) and 2.896(1) Å), where the intermetallic contacts are supported by the P,S-donor bridging ligands.^19^ These Au–Pb distances are also much shorter than those reported in other unsupported Au···Pb systems by other research groups (3.6998(14) Å).^22^

In these structures, the trinuclear pieces are held together by aurophilic interactions that give rise to extended chains in the cases of complexes **2** and **3** (Figures 2 and 3, respectively), while in the structure of **4,** only two of these Au_2_Pb units are linked through a unique Au···Au interaction (Figure 4), that is, the lower the solvent content, the higher the nuclearity. Moreover, there is a relationship between the nuclearity and the strength of the aurophilic interactions in these three crystal structures, since the polymeric compounds display shorter Au–Au distances, of 3.0391(6) Å in **2**, and of 3.029(4) and 2.962(3) Å in **3**, than the distance in the hexanuclear complex, of 3.3386(15) Å. In contrast, this trend is not observed in the related terpyridine derivatives mentioned above, in which the Au–Au distances range from 3.5413(7) to 2.8920(5) Å, and no relation is found between the length of the intermetallic chain and the strength of the aurophilic interaction.^21,23^

**Figure 2:**
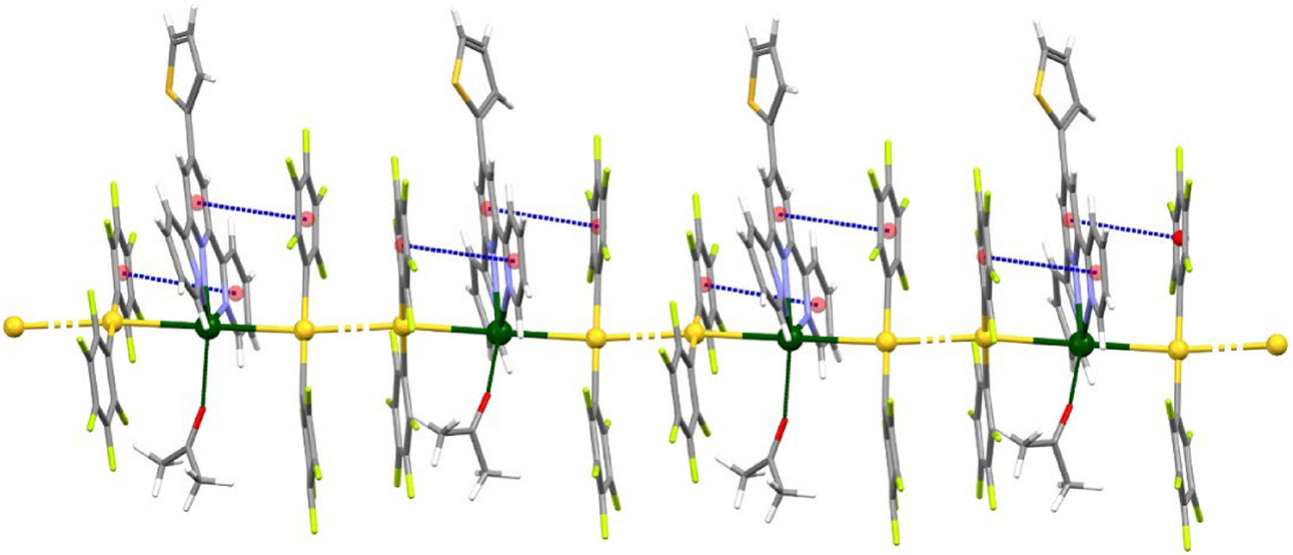
Partial view of the polymeric chain in the crystal structure of complex **2**. Color code: gold (yellow), lead (green), oxygen (red), nitrogen (purple), sulfur (orange), carbon (gray), fluorine (light green), and π–π interactions (dashed blue).

**Figure 3:**
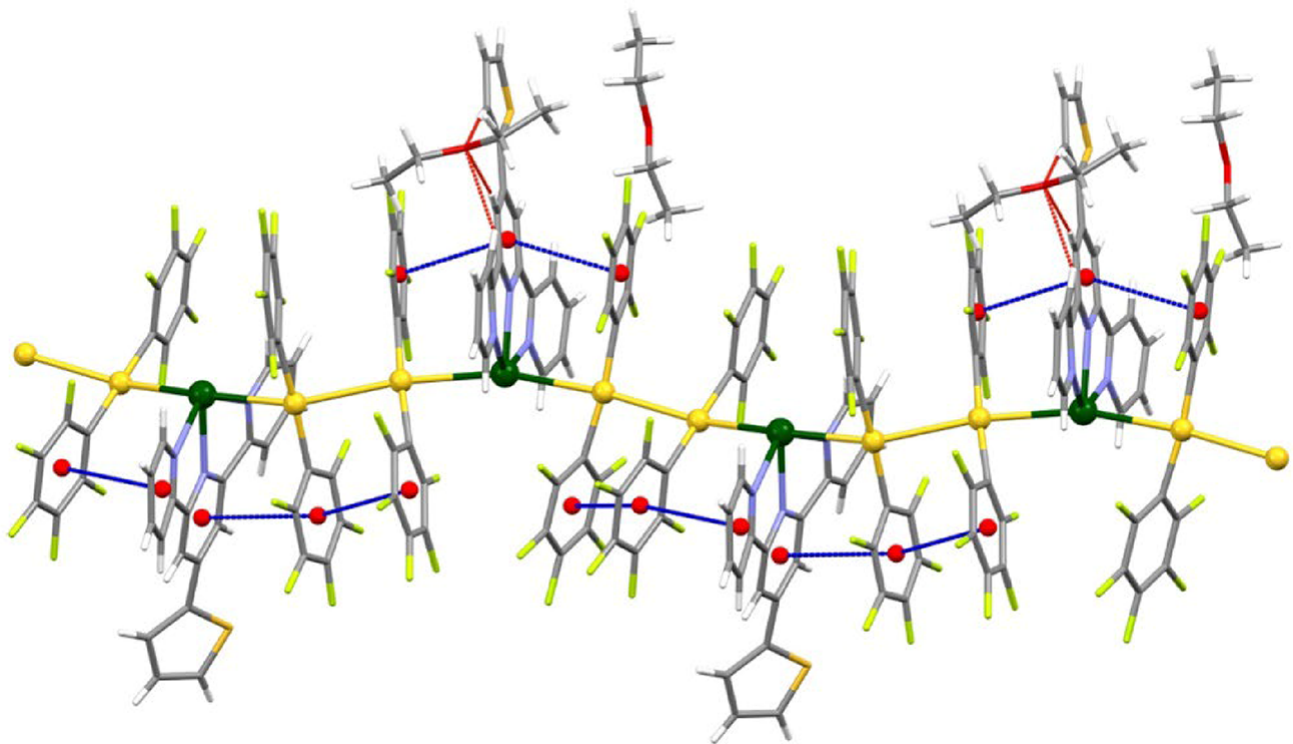
Partial view of the polymeric chain in the crystal structure of complex **3**. Color code: gold (yellow), lead (green), oxygen (red), nitrogen (purple), sulfur (orange), carbon (gray), fluorine (light green), π–π interactions (dashed blue), and C–H···O (dashed red) hydrogen bonds.

**Figure 4:**
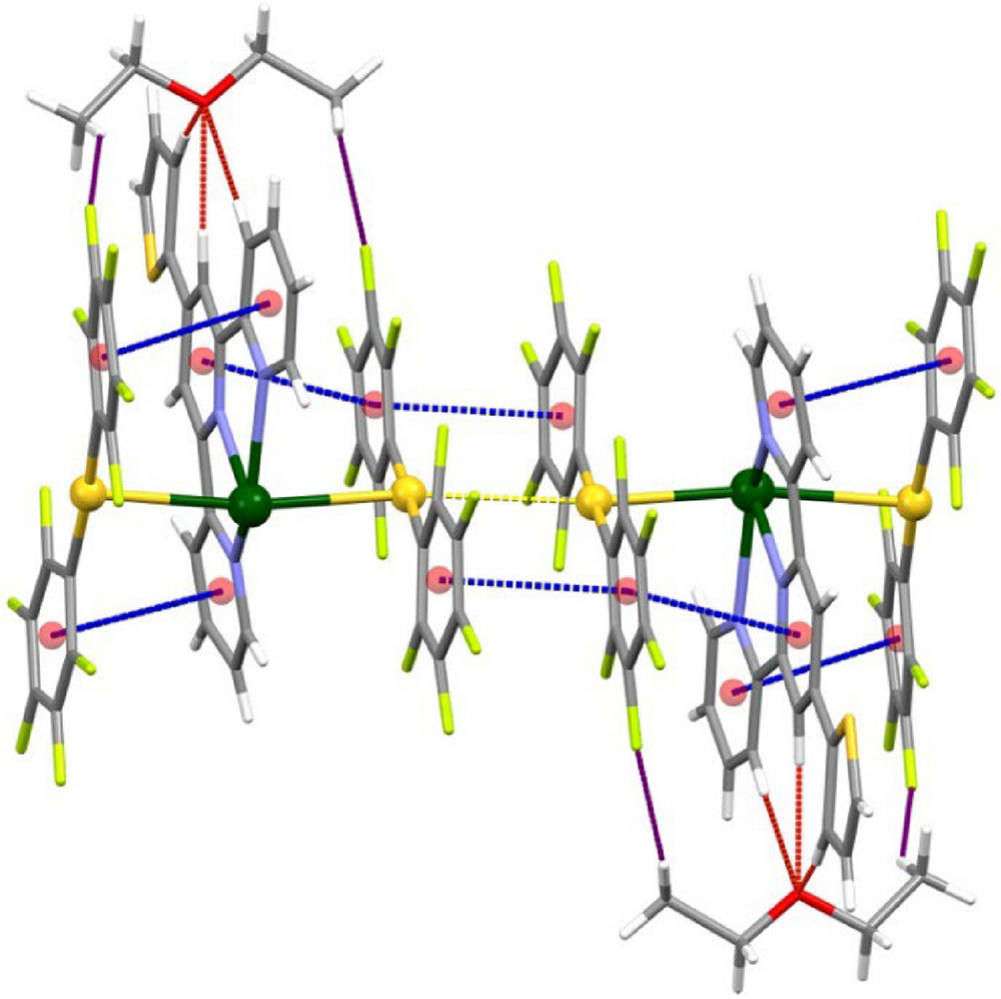
Crystal structure of complex **4**. Color code: gold (yellow), lead (green), oxygen (red), nitrogen (purple), sulfur (orange), carbon (gray), fluorine (light green), π–π interactions (dashed blue), C–H···O (dashed red), and C–H···F (dashed purple) hydrogen bonds.

As can be observed in Figures 2 and 3, as well as inferred from the data in Table 1, apart from the solvent, the main differences between the polymeric structures of **2** and **3** lies in the disposition of the S-Terpy ligands (that are oriented in the same direction throughout the polymer in **2**, while appear in an alternated fashion in **3**) and in the Au–Pb–Au and Pb–Au–Au angles along the heterometallic chain (that in **2** are approximately linear, while in **3** are much narrower, with a minimum of 149.24(8)° in one of the Pb–Au–Au angles).

**Table 1:** Selected Bond Distances (Å) and Angles (°) in the Crystal Structures of **2–4**

	Au··Pb	Au···Au	Pb–N	Au–Pb–Au	Pb–Au–Au	C–Au–C	N–Pb–N
**2**	2.9776(7)	3.0391(6)	2.534(10)	179.42(2)	172.90(2)	179.2(5)	66.0(3)
	2.9578(7)		2.500(8)		172.76(2)	177.9(5)	64.6(3)
			2.477(9)				
**3**	2.917(4)						
	2.879(3)	3.029(4)	2.52(3)-	168.94(6)	172.04(6)-	175.4(12)-	68.1(8)-
	2.869(3)	2.962(3)	–2.32(2)	160.69(7)	–149.24(8)	–173.5(8)	–64.3(8)
	2.856(4)						
**4**	2.9639(9)	3.3386(15)	2.462(17)	170.26(3)	161.66(4)	176.3(9)	65.7(5)
	2.9642(10)		2.456(13)			175.0(7)	65.9(5)
			2.452(16)				

Finaly, the hexanuclear dimer formed by two Au_2_Pb units in **4** further interacts with two adjacent dimers via weak Pb···F contacts of 3.448(11) and 3.492(11) Å as well as through weak Au···F interactions of 3.377(14) Å in which different perhalophenyl groups of the (Au–Pb–Au)_2_ hexanuclear unit are involved (van der Waals radii: Pb, 2.60; Au, 2.32; F, 1.46 Å).^26^ The presence of these metal···halogen contacts results in the formation of a one-dimensional chain that runs along the [1 0 0] direction, as shown in Figure 5.

**Figure 5:**
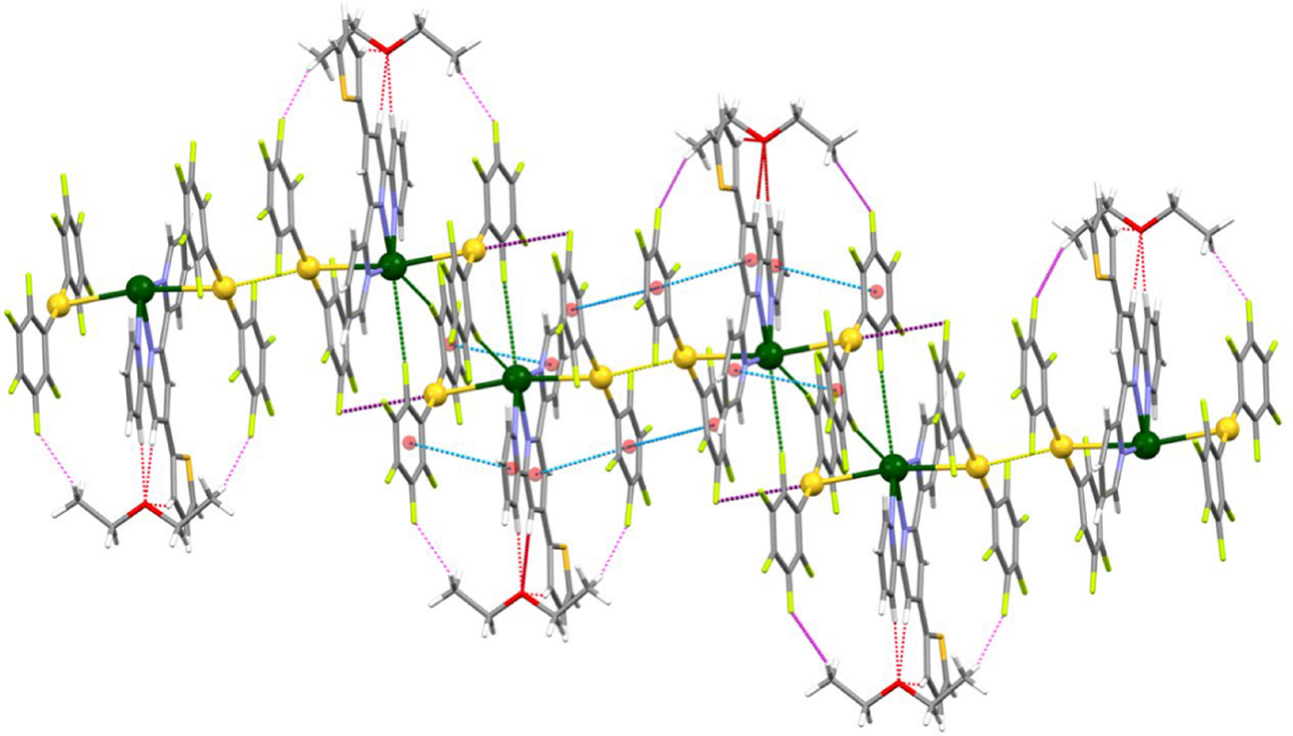
Partial view of the 1D network in the crystal structure of complex **4**. Color code: gold (yellow), lead (green), oxygen (red), nitrogen (purple), sulfur (orange), carbon (gray), fluorine (light green), π–π interactions (dashed blue), C–H···O (dashed red), and C–H···F (dashed pink) hydrogen bonds, Pb···F (dashed green), and Au···F (dashed purple) contacts.

With respect to the metal environments within the Au–Pb–Au building blocks, in all cases, the gold­(I) centers are linearly coordinated to two pentafluorophenyl rings with Au–C bond lengths between 1.938(16) Å in **3** and 2.047(12) Å in **2**. The aryl rings within each bis­(aryl)­aurate­(I) anion are not far from planarity except in the case of **3**, where angles between 12.8 and 20.9° are found. Regarding the cationic {Pb­(S-terpy)}^2+^ fragment, the S-Terpy ligand is coordinated to lead in a tridentate chelating mode, showing similar Pb–N bond distances in the cases of compounds **2** (2.534(10), 2.500(8) and 2.477(9) Å) and **4** (2.462(17), 2.456(13) and 2.452(16) Å), while in the crystal structure of **3** the Pb–N bond distances are more dissimilar (2.52(3)–2.32(2) Å). Nevertheless, most of them lie within the usual values found in other lead­(II) derivatives containing terpy-type ligands (2.568(3)–2.450(7) Å).^20a,21,23,24^

On the other hand, it is worth noting that the three structures display different coordination numbers for the lead centers and/or environment (Figure 6). In complex **2**, each acetone molecule is connected to a lead atom through a Pb···O contact of 2.864(17) Å, which leads to a coordination number of 6. As shown in Figure 6 left, the lead atom in **2** shows a distorted pentagonal bipyramidal environment in which the gold centers take up the apical positions, while the stereochemically active lead­(II) lone pair occupies one of the equatorial ones. On the contrary, the solvent molecules do not interact directly with the lead atoms in the ether derivatives **3** and **4**, but they link up the polymeric chain via C–H···O (**3**) or C–H···O and C–H···F hydrogen bonds (**4**) (see Figure 1). This would make us expect a coordination number of 5 for lead­(II) in these two structures; nevertheless, this is true only in **3** (Figure 6 center), where, as in **2**, the lead­(II) lone pair seems to be stereochemically active, leading to a hemidirected environment for this metal center.^25^ In contrast, the above-mentioned Pb···F interactions present in the supramolecular structure of **4** increase the coordination number of lead to 7. In this case, the metal displays a holodirected environment and a very distorted pentagonal bipyramidal geometry, with the three nitrogen atoms of an S-Terpy and two fluorine centers occupying the equatorial positions (Figure 6 right).

**Figure 6:**
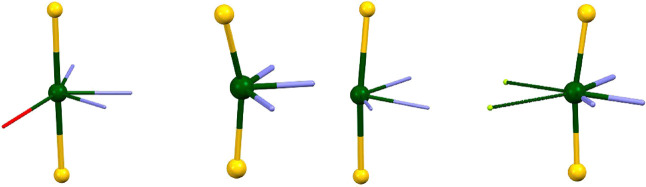
Coordination environments for the lead­(II) atoms in the crystal structures of complexes **2** (left), **3** (center), and **4** (right). Color code: gold (yellow), lead (green), oxygen (red), nitrogen (purple), and fluorine (light green).

Despite the differences in the coordination numbers and environments, the N–Pb–N angles are comparable in all compounds (see Table 1), which is probably due to the rigidity of the tridentate ligand, whereas the Au–Pb–Au angles differ significantly, being nearly linear in **2** and with values of 168.94(6) and 160.69(3)° in **3**, and of 170.26(3)° in **4** (see Figure 6).

Finally, in all three structures, the Au···Pb interactions are reinforced by π–stacking between one aryl ring of each anionic gold fragment and a pyridyl ring of the central S-Terpy ligand (Figure 1). Moreover, in the cases of **3** and **4,** π-stacking between pentafluorophenyl rings also enhances the Au···Au interactions (Figures 3 and 4). All these interactions, though varying in strength, show centroid-to-centroid distances within the range typically associated with π-stacking interactions.^26^

### Optical Properties of the Complexes

The absorption spectra in solution confirm the previously discussed dissociation process that may occur in solution. Thus, the spectra of all four compounds are very similar, displaying a band at 263 nm that appears in the gold precursor, NBu_4_[Au­(C_6_F_5_)_2_], which is associated with an internal π–π transition between the π orbitals of the perhalophenyl groups or, alternatively, with a charge transfer between the gold center and these π orbitals. Additionally, a second band appears in all the spectra around 288 nm, which is attributed to internal π–π transitions of the aromatic rings in the S-Terpy ligand, since this free ligand possesses a similar absorption (see Figure 7). The observed differences in absorption intensities result from the noninnocent role of the incorporated solvents. During the dissociation process in solution, not all Pb–S–Terpy fragments are equivalent, leading to variations in their UV–vis spectra.

**Figure 7:**
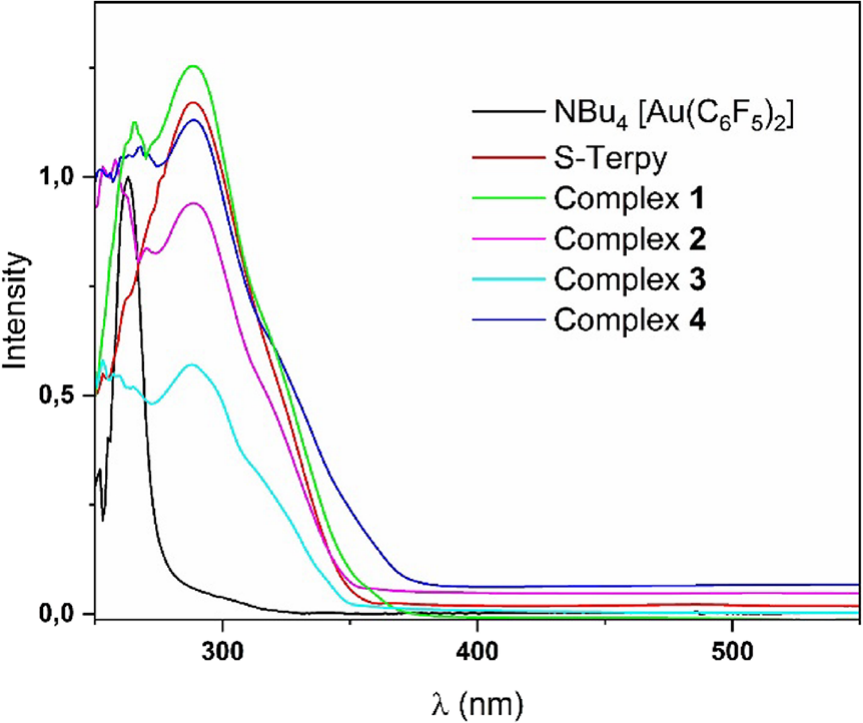
Experimental UV–vis spectra in DMSO solution of complex **1** (3.03 × 10^–5^ M), complex **2** (2.75 × 10^–5^ M), and complex **3** (3.15 × 10^–5^ M) and complex **4** (2.95 × 10^–5^ M).

By contrast, in the solid state absorption spectra, in addition to these high energy bands between 260 and 350 nm that are likely to correspond to π–π or charge transfer transitions involving the C_6_F_5_ and S-Terpy rings, additional lower energy bands of low intensity appear in the 500–700 nm region. These bands are considerably more intense in the case of complex **3**, which suggests an allowed nature for them in this particular case. On the other hand, the existence of these low energy bands in the solid state spectra of the four complexes that do not appear in their solution spectra suggests that these transitions may involve gold–gold and/or gold–lead interactions (Figure 8).

**Figure 8:**
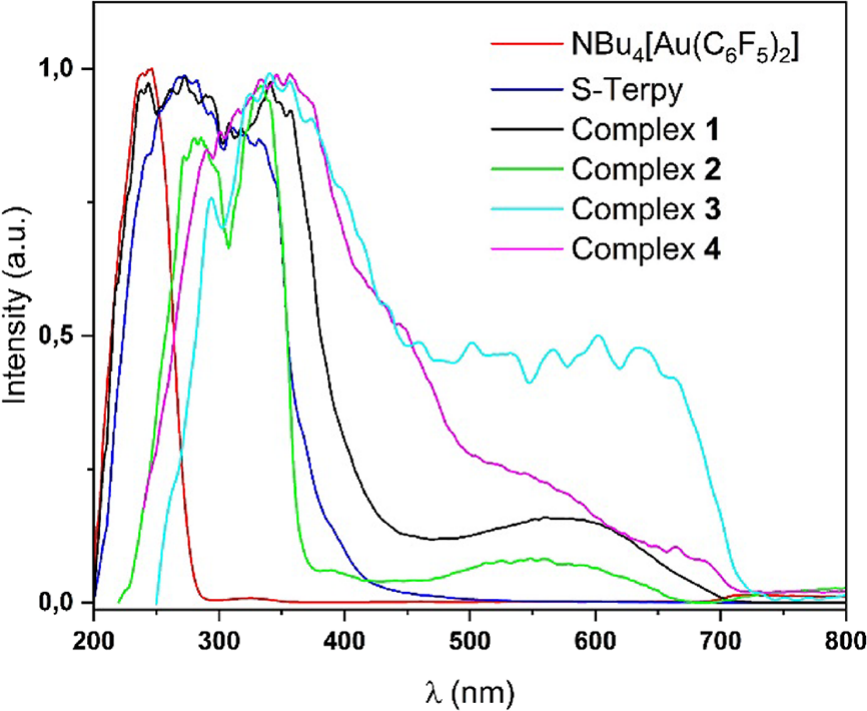
Experimental UV–vis solid state absorption spectra for complexes **1–4**, S-Terpy (red), and gold precursor.

The four complexes show intense energy emissions at room temperature and at 77 K in the solid state (Figure 9). Complex **1** emits at 702 nm (excitation at 600 nm), complex **2** at 732 nm (excitation at 600 nm), complex **3** at 750 nm (excitation at 690 nm), and complex **4** at 557 nm (excitation at 440 nm). These emissions shift to 810 nm (excitation at 632 nm), 846 nm (excitation at 670 nm), 900 nm (excitation at 730 nm), and 565 nm (excitation at 435 nm) when measurements are carried out at 77 K. This shift to lower energies when decreasing the temperature, which is observed for all the complexes, is likely to be related to a reduced HOMO–LUMO gap, resulting from the shortening of interatomic distances by thermal contractions at low temperature.^26^ In addition, in the case of complexes **2** and **3**, whose structures are built by an infinite sequence of repeating gold–lead-gold units, we can observe different optical behaviors. In the case of complexes **1** and **2**, the excitation spectra match the less energetic zone of the absorption spectra, where the intensities of the bands are much weaker, indicating their forbidden nature. This is further confirmed by their lifetime measurements, which are in the microsecond range. By contrast, complex **3** shows a lifetime of 53 ns, indicating that the emission is a fluorescent process. In addition, as can be observed in Table 2, the values obtained for the lifetimes at 77 K for complexes **1**, **2**, and **3** are higher than those obtained at room temperature, indicating a reduction of the nonradiative constants (*k*_nr_) at low temperatures due to the decrease in molecular motions. The coincidence in the spectral region in which these three complexes emit (infrared) suggests that they display similar structures; therefore, according to these data, we propose a similar polymeric nature for complex **1**.

**Figure 9:**
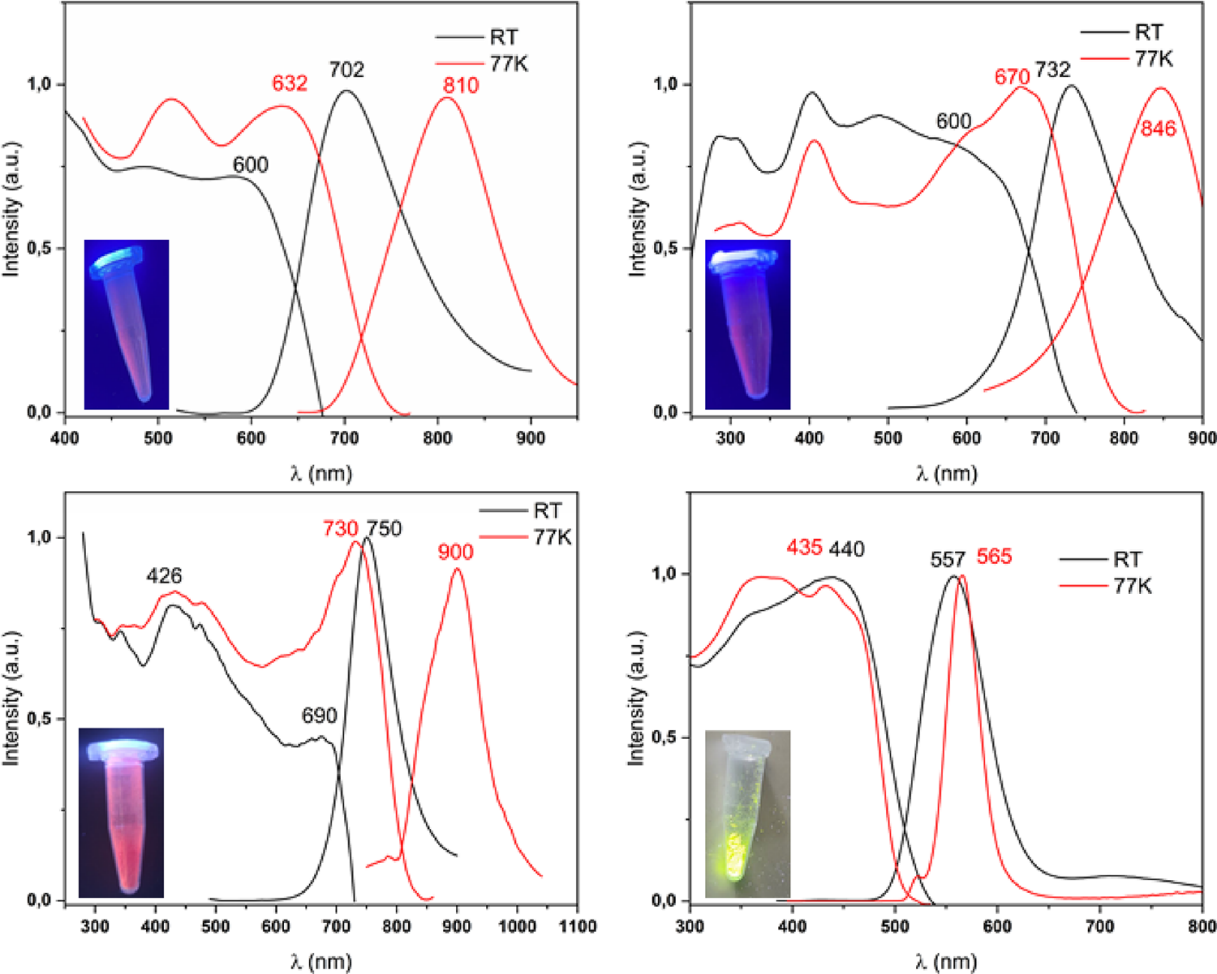
Excitation and emission spectra in the solid state at RT and 77 K, complex **1** (top left), complex **2** (top right), complex **3** (lower left), and complex **4** (lower right).

**Table 2:** Photophysical Properties of Complexes **1–4**

complex	1	2	3	4
Solid em(ex) (RT)	702 (370–600)	732 (240–600)	750(280–690)	557 (280–440)
Solid em (ex) (77 K)	810 (420–632)	846 (280–670)	900(300–750)	565 (280–435)
τ (RT) μs	2.3715	3.374	0.053	33.74
τ (77 K) μs	3.3518	12.14	2.764	12.14
Φ (RT)	3.7	2.3	3.3	17.7

In this type of structure, in which metal···metal interactions are especially important in their emissive behavior, it is common that the shorter the intermetallic interactions are, the lower energy wavelengths are observed. In fact, this occurs between the isostructural complexes **2** and **3**, the latter showing shorter interatomic distances (see X-ray Crystal Structure Determination section). Following this reasoning, and assuming a similar structure for complex **1**, we propose longer intermetallic distances in the nonsolvated derivative. It can also be observed that the emission maxima for complexes **1–3**, both at room temperature and at 77 K, are shifted to lower energies compared to other gold–lead systems previously reported by our group.^20a^

The large Stokes shifts, along with the measured lifetimes both at room temperature and at 77 K, which are on the order of microseconds for complexes **1**, **2**, and **4**, suggest that these processes may be attributed to phosphorescence. However, in the case of compound **3**, a shorter excited-state lifetime is observed at RT compared with the previous compounds. Additionally, its solid state absorption spectrum matches the excitation spectrum, suggesting a fluorescent behavior. Very interestingly, in the case of compound **4**, whose structure is different, consisting of six-metal clusters that polymerize by π–π interactions, a decrease in the lifetime at 77 K compared to that measured at room temperature is observed. This behavior may indicate that the emission at both temperatures originates from different triplet states (Figure 10). Finally, quantum yield measurements of complexes **1**–**4** in the solid state at room temperature exhibit values of 3.7% (**1**), 3.3% (**2**), 3.3% (**3**), and 17.7% (**4**).

**Figure 10:**
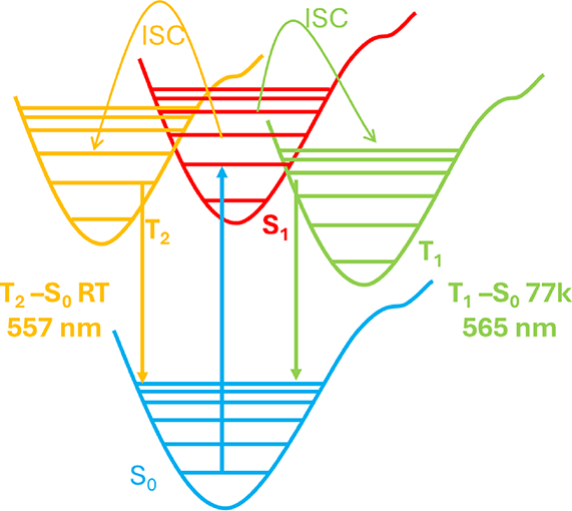
Emission states for complex **4** at RT and 77 K.

### Computational Studies

Single-point DFT and TD-DFT calculations were performed to explain the origin of the emission of the complexes based on their solid state structure. Models were chosen based on the crystal structures of complexes **2**, **3**, and **4**. Models systems for **2**–**4** were built up considering two equal Au_2_Pb units linked through an unsupported gold­(I)···gold­(I) contact to study these types of interactions. Regarding compound **1**, the X-ray structure of compound **2** was used, with the solvent removed, and the structure was optimized.

For compound **1**, as can be seen in Table S1, the HOMO is almost entirely centered on the S-Terpy ligand, whereas in compounds **2**, **3**, and **4** the HOMO is mainly localized on the gold centers, with some contribution from the pentafluorophenyl rings. On the other hand, the LUMO in all models is mainly localized on the S-Terpy ligand and exhibits an antibonding π character.

This suggests that the N-donor ligand plays a significant role in the luminescent behavior, being involved in metal-to-ligand charge transfer (MLCT) transitions in the case of compounds **2**, **3**, and **4**.

We computed the first 50 singlet–singlet excitations for model systems **1**–**4** at the TD-DFT level of theory (Tables 3–6). In addition, we computed the lowest energy singlet–triplet excitation for models **1**, **2**, and **4** to represent the origin of their phosphorescent behavior. We compared the corresponding predicted energies with the experimental excitation spectra.

**Table 3:** TD-DFT First Singlet–Singlet and Lowest Singlet-Triplet Excitation (Exc) Wavelengths (λ_Calc_) and Oscillator Strengths (*f*) Calculated for Model **1**

complex	exc	λ_Calc_ (nm)	*f*	contributions
**1**	S_0_ → T_1_	668.88		HOMO – 6 → LUMO (14%)
				HOMO – 5 → LUMO (13%)
				HOMO – 3 → LUMO (14%)
	S_0_ → S_9_	693.45	0.0364	HOMO – 10 → LUMO (19%)
				HOMO – 7 → LUMO (16%
				HOMO → LUMO + 1 (37%)
	S_0_ → S_18_	631.67	0.0515	HOMO → LUMO + 3 (65%)
	S_0_ → S_34_	433.41	0.2837	HOMO – 1 → LUMO + 1 (74%)

**4:** TD-DFT First Singlet–Singlet and Lowest Singlet-Triplet Excitation (Exc) Wavelengths (λ_Calc_) and Oscillator Strengths (*f*) Calculated for Complex **2**

complex	exc	λ_Calc_ (nm)	*f*	contributions
**2**	S_0_ → T_1_	523.43		HOMO → LUMO (87%)
	S_0_ → S_1_	487.58	0.1375	HOMO → LUMO (96%)
	S_0_ → S_21_	382.19	0.0433	HOMO – 7 → LUMO + 1 (29%)
				HOMO – 14 → LUMO + 1 (26%)
	S_0_ → S_30_	370.26	0.0617	HOMO – 14 → LUMO + 1 (21%)
				HOMO – 7 → LUMO + 1 (19%)
				HOMO – 8 → LUMO + 1 (12%)

**5:** TD-DFT First Singlet–Singlet, Wavelengths (λ_Calc_), and Oscillator Strengths (*f*) Calculated for Complex **3**

complex	exc	λ_Calc_ (nm)	*f*	contributions
**3**	S_0_ → S_1_	503.57	0.2424	HOMO → LUMO (98%)
	S_0_ → S_22_	349.07	0.0785	HOMO – 9 → LUMO (22%)
				HOMO – 11 → LUMO (18%)
				HOMO – 4 → LUMO + 1 (10%)
	S_0_ → S_24_	385.93	0.0532	HOMO – 14 → LUMO (34%)
				HOMO – 6 → LUMO + 1 (30%)

**Table 6:** TD-DFT First Singlet–Singlet and Lowest Singlet-Triplet Excitation (Exc) Wavelengths (λ_Calc_) and Oscillator Strengths (*f*) Calculated for Complex **4**

complex	exc	λ_Calc_ (nm)	*f*	contributions
**4**	S_0_ → T_1_	525.02		HOMO – 14 → LUMO (5%)
				HOMO – 4 → LUMO + 1 (6%)
				HOMO – 5 → LUMO (10%)
				HOMO – 4 → LUMO (12%)
				HOMO – 2 → LUMO (12%)
	S_0_ → T_2_	523.84		HOMO – 4 → LUMO + 1 (14%)
				HOMO – 5 → LUMO + 1 (13%)
				HOMO – 4 → LUMO (8%)
				HOMO – 7 → LUMO + 1 (6%)
	S_0_ → S_4_	438.15	0.0720	HOMO → LUMO + 1 (55%)

	S_0_ → S_25_	376.97	0.0873	HOMO – 14 → LUMO + 1 (37%)
				HOMO → LUMO + 4 (23%)
	S_0_ → S_50_	341.85	0.2619	HOMO → LUMO + 5 (48%)HOMO → LUMO + 1 (55%)
				HOMO – 14 → LUMO (11%)

The analysis of the most intense (in terms of oscillator strength) singlet–singlet transitions for complex **1** shows electronic excitations between 705 and 306 nm, the most intense excitation wavelengths appearing at 693, 631, and 433 nm. The less energetic of these singlet–singlet transitions at 693 nm would arise from a transition from the perhalophenyl rings bonded to the gold centers to the S-Terpy ligand coordinated to lead, representing a ligand-to-ligand charge transfer transition (LLCT), with some contribution from an S-Terpy-based intraligand transition (IL). The electronic excitation computed at 631 nm takes place between molecular orbitals placed on the S-Terpy ligand, indicating an intraligand transition (IL). Finally, the computed excitation at 433 nm would involve an occupied MO located at the gold metal centers and an empty MO placed on the S-Terpy ligand, leading to a metal-to-ligand charge transfer (MLCT) transition.

In the case of the model system of complex **2**, the computed singlet–singlet excitations appear in a higher energy range, between 487 and 370 nm, compared to complex **1**, and are in agreement with its solid state absorption spectrum. The most intense transitions are observed at 487, 382, and 370 nm. The lowest energy transition involves the occupied gold-based HOMO and the empty S-Terpy ligand LUMO, in a metal-to-ligand charge transfer transition (MLCT). The electronic excitations at 382 and at 370 nm display a similar origin, corresponding to a transition between MOs located on the pentafluorophenyl ligand to MOs with a main S-Terpy composition, leading to ligand-to-ligand charge transfer transitions (LLCT).

For compound **3**, the calculated singlet–singlet transitions appear within the range of 503 to 386 nm. The three most intense excitations are observed at 503, 349, and 386 nm. The lowest-energy transition corresponds to a metal-to-ligand charge transfer (MLCT) from the MOs located on the gold metal centers to virtual MOs on the S-Terpy ligands. In contrast, the two high energy transitions originate from the pentafluorophenyl ligand to the S-Terpy ligand and can be described as ligand-to-ligand charge transfer transitions (LLCT).

Finally, for compound **4**, the calculated singlet–singlet transitions are located within the range of 441 to 342 nm, being more energetic compared to the previous ones and in agreement with the absorption spectrum obtained for this complex. The three most intense transitions are observed at 438, 376, and 342 nm. The analysis of the MOs involved in these three electronic transitions indicates that all of them display similar contributions, in agreement with excitations from gold-based MOs to S-Terpy-based MOs in metal-to-ligand charge transfer transitions (MLCT).

After the analysis of the most intense allowed singlet–singlet excitations, we also studied the nature of the first singlet–triplet electronic excitations, related to the phosphorescent behavior of complexes **1**, **2**, and **4**. In the case of complex **1**, the lowest singlet–triplet transition displays a mixed contribution from excitations between occupied HOMO – 6, HOMO – 5, and HOMO – 3 orbitals to the LUMO, all of them related to metal (gold) to ligand (S-Terpy) charge transfer transitions (MLCT). In the case of complex **2**, the lowest singlet–triplet transition arises from the HOMO and arrives at the LUMO, displaying, again, a similar MLCT origin.

As previously commented, the case of compound **4** is different since at 77 K, the emission arises from a different triplet state to the one found at RT. Accordingly, the first two lowest singlet–triplet excitations were computed. The low energy singlet–triplet excitation at 525 nm originates from a mixture of several electronic excitations between the occupied HOMO – 14, HOMO – 5, HOMO – 4, and HOMO – 2 to LUMO and LUMO + 1 and displays a ligand-to-ligand charge transfer character (LLCT) occurring from the pentafluorophenyl ligands to the S-Terpy ligands. In contrast, the higher energy singlet–triplet excitation at 523 nm arises from a mixture of electronic transitions between HOMO – 7, HOMO – 5, and HOMO – 4 and LUMO and LUMO + 1, exhibiting a similar LLCT character as in the previous case. Although the main contribution to both singlet–triplet transitions arises from LLCT, in the case of the S_0_-T_1_ one, there is a minor but not negligible participation of the gold centers in HOMO – 14 and HOMO – 2, this transition being better described as a metal-perturbed LLCT.

As previously mentioned, complex **4** is not a linear chain with aurophilic interactions, as observed in compounds **1**–**3**. Instead, it features Pb···F interactions. These interactions result in lead adopting a pentagonal bipyramidal geometry. Consequently, the lone pair is less pronounced compared to compound **1**, as evidenced by electron localization function (ELF) calculations. In compound **1**, the lone pair appears slightly more prominent than in compound **4** (see Figure 11).

**Figure 11:**
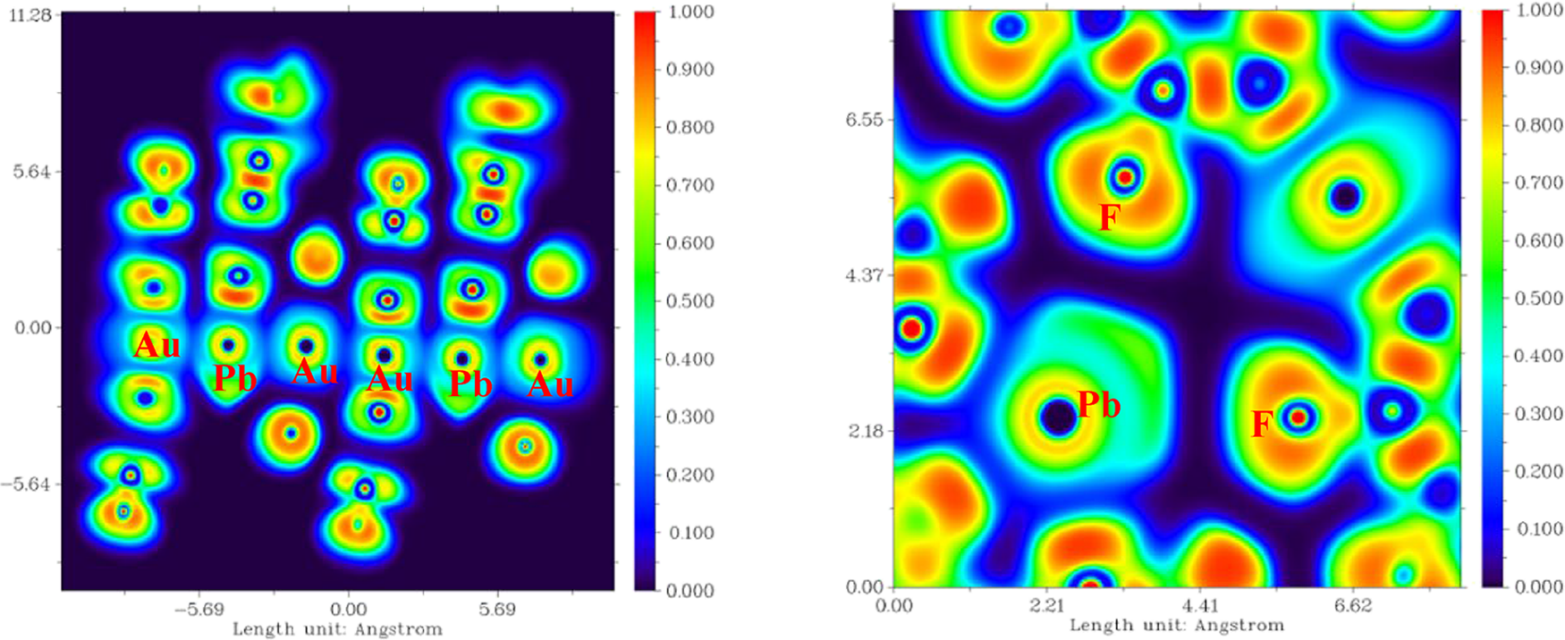
Two-dimensional (2D) ELF plots for complexes **1** (left) and **4** (right).

## Conclusions

Compound **1** displays pronounced solvatochromic behavior and is capable of incorporating organic molecules into its crystal lattice. This host–guest interaction induces not only structural and color modifications but also significant changes in luminescence. Theoretical analyses further reveal distinct photophysical origins across the series: in compounds **1** and **2**, the emission is mainly attributed to a forbidden metal-to-metal ligand charge transfer (MMCT) process, whereas in compound **4**, the dominant pathway involves ligand-to-ligand charge transfer (LLCT). The structural variations between compounds **1**–**3** and **4** thus account for the observed sharp luminescence shift with emission changing from red to yellow.

Importantly, the observation and assignment of such changes in emission—from MLCT/MMCT to LLCT— not only are crucial for understanding the intrinsic properties of these systems but also highlight a phenomenon that is probably more common in supramolecular assemblies than has generally been recognized. This work therefore emphasizes the need to carefully consider charge-transfer contributions when analyzing luminescence in related coordination and supramolecular systems.

Overall, these findings underscore the sensitivity of emission properties to subtle structural variations and guest inclusion effects, providing valuable insights for the rational design of new luminescent supramolecular materials with tunable photophysical responses.

## Experimental Section

### General

[Au_2_Ag_2_(C_6_F_5_)_4_(Et_2_O)_2_]_*n*_ was prepared according to literature methods.^18a^

### Materials and Physical Measurements

Infrared spectra were recorded in the 3500–450 cm^–1^ range on a PerkinElmer FT-IR Spectrum Two with a UATR (Single Reflection Diamond) accessory. ^1^H and ^19^F NMR spectra were recorded on a Bruker Avance 400 instrument in deuterated DMSO solutions at room temperature. Chemical shifts are quoted relative to those of SiMe_4_ (^1^H external) and CFCl_3_ (^19^F external). C, H, and N analyses were carried out with a C.E. Instrument EA-1110 CHNSO microanalyzer. The MALDI mass spectra were registered on a Microflex Bruker spectrometer using DIT (dithranol) and DCTB (T-2-(3-(4-*t*-butylphenyl)-2-methyl-2-propenylidene)-malononitrile) as the matrix. The *m*/*z* values are obtained for the higher peak in the isotopic pattern. Absorption spectra in solution were recorded on a Hewlett-Packard 8453 diode array UV–visible spectrophotometer. Diffuse reflectance UV–vis spectra of pressed powder samples diluted with silica were recorded on a Shimadzu UV-3600 spectrophotometer with a Harrick Praying Mantis accessory and recalculated following the Kubelka–Munk function. Excitation and emission spectra and luminescence lifetimes in the solid state were recorded with an Edinburgh FLS 1000 fluorescence spectrometer. Time-resolved decay measurements were analyzed using numerical deconvolution. To do this, the corresponding instrumental response function (IRF) is necessary to detect the scattered light from the sample. Quantum yields were measured in the solid state using a Hamamatsu Quantaurus-QY C11347–11 integrating sphere.

### Synthesis

The S-Terpy ligand was synthesized according to the reported literature method, and the experimental details of its synthesis are consistent with those previously described.^27,28^

#### [{Au­(C_6_F_5_)_2_}_2_{Pb­(S-Terpy)}]_*n*_ (**1**)

To a solution of [Au_2_Ag_2_(C_6_F_5_)_4_(OEt_2_)_2_]_*n*_ (0.142 g, 0.1 mmol) in methanol (30 mL) was added PbCl_2_ (0.028 g, 0.1 mmol), and a white precipitate (AgCl) was formed. The reaction mixture was stirred at room temperature for 3 h and then filtered. Then, it was added to the previous mixture S-Terpy (0.031 g, 0.1 mmol). The reaction mixture was stirred at room temperature for 2 h. Total evaporation of the solvent under vacuum gave rise to complex **1** as a red solid. Yield 80% (0.081 g). ^1^H NMR (400 MHz, [D_6_]-DMSO, ppm): δ 8.71­[d, 2H, H_1_] [^3^ *J*_H1–H4_ = 4 Hz], 8.64–8.62 [m, 4H, H_2_+H_3_], 8.06–8.04 [m, 2H, H_4_], 8.02 [m, 1H, H_5_], 7.85–7.84 [m, 1H, H_9_], 7.55­[m, 2H, H_7_], 7.30 [m, 1H, H_8_] [(^3^ *J*_H8–H5_) ∼ (^3^ *J*_H8–H9_) = 4.28 Hz]. ^19^F NMR (376.752 MHz, [D_6_]-DMSO, ppm): δ −162.81 [m,4F, F_m_], −161.47 [t, 2F, F_p_] [^3^ *J*_p‑m_ = 24 Hz], −114.52 [m, 4F, F_o_]. FT-IR­(UATR): ν = 1600 cm^–1^ (C=N), ν = 1450 cm^–1^ (C=C) and ν = 1499, 948, and 782 cm^–1^ (Au–C_6_F_5_). MALDI (+) *m*/*z* (%): 1054,066 (89.11) [Au­{C_6_F_5_}_2_Pb–S-terpy]^+^. MALDI (−) *m*/*z* (%): 530.879 (100) [AuC_6_F_5_]^−^. Elemental analysis calcd (%) for Au_2_PbN_3_SC_43_F_20_H_13_: C, 32.59; H,0.83; N, 2.65; S, 2.02. Found C, 32.73; H, 0.79; N, 2.52; S, 1.95. Λ_m_ (methanol): 70 Ω^–1^ cm^2^ mol^–1^.

#### {[{Au­(C_6_F_5_)_2_}_2_{Pb­(S-Terpy)}]·0.5Me_2_CO}_*n*_ (**2**)

A solution of compound **1** (0.200 g, 0.1262 mmol) in acetone (10 mL) was stirred for 10 min. Evaporation of the solvent to dryness gave a red solid in almost quantitative yield. ^1^H NMR (400 MHz, [D_6_]-DMSO, 298 K): δ 8.78 [pd,2H, H_1_] [^3^ *J*_H1–H4_ = 4 Hz], 8.66–8.65 [m,4H, H_2_ + H_3_], 8.04­[pt,2H, H_4_] [(^3^ *J*_H4–H1_) ∼ (^3^ *J*_H4–H7_) = 10 Hz], 7.99 [d,1H, H_5_] [^3^ *J*_H5–H8_ = 3.2 Hz], 7.82 7.81 [m,1H, H_9_], 7.55 [m,2H, H_7_], 7.28 [pt,1H, H_8_] [(^3^ *J*_H8–H9_) ∼ (^3^ *J*_H8–H5_) = 4 Hz], 2.05 [m,6H, CH_3_]. ^19^F NMR (376.752 MHz, [D_6_]-DMSO, 298 K): δ −162.82 [m, 4F, F_m_], −161.5 [t, 2F, F_p_] [^3^ *J*_p‑m_ = 24 Hz], −114.56 [m, 4F, F_o_]. FT-IR (UATR) *v* = 1700 cm^–1^ (C=O), 1605 cm^–1^ (C=N), 1449 cm^–1^ (C=C), 787, 951, and 1499 cm^–1^ (Au–C_6_F_5_). MALDI (+) *m*/*z* (%): 1054.066 (89.11) [Au­{C_6_F_5_}_2_Pb–S-terpy]^+^. MALDI (−) *m*/*z* (%): 530.879 (100) [AuC_6_F_5_]^−^. Elemental analysis calcd (%) for Au_2_PbN_3_SC_46_F_20_H_19_O: C, 33.63; H,1.17; N, 2.56; S,1.95. Found C, 33.02; H, 1.12; N, 2.44; S, 2.05. Λ_m_ (methanol): 54 Ω^–1^ cm^2^ mol^–1^.

#### {[{Au­(C_6_F_5_)_2_}_2_{Pb­(S-Terpy)}]·0.5Et_2_O}_*n*_ (**3**)

A solution of compound **1** (0.200 g, 1262 mmol) in diethyl ether (10 mL) was stirred for 10 min. Evaporation of the solvent to dryness gave a red solid in almost quantitative yield. ^1^H NMR (400 MHz, [D_6_]-DMSO, 298 K): δ 8.79 [d,2H,H_1_] [^3^ *J*_H1–H4_ = 4 Hz], 8.68–8.66 [m. 4H,H_2_ + H_3_], 8.05 [pt, 2H, H_4_] [(^3^ *J*_H4–H1_) ∼ (^3^ *J*_H4–H7_) = 7.2] Hz, 8.00 [d,1H, H_5_] [^3^ *J*_H5–H8_ = 3.4 Hz], 7.84–7.82 [m, 1H, H_9_], 7.55 [m, 2H, H_7_], 7.30 [pt, 1H, H_8_] [(^3^ *J*_H8–H5_) ∼ (^3^ *J*_H8–H9_) = 4 Hz], 0.92 [t,6H,CH_3_] [^3^ *J*_H(CH3)‑H(CH2)_] = 8 Hz]. ^19^F NMR (376.752 MHz, [D_6_]-DMSO, ppm): δ −162.82 [m,4F, F_m_], −161.49 [t, 2F, F_p_] [^3^ *J*_p‑m_ = 24 Hz], −114.55 [m, 4F, F_o_]. FT-IR (UATR) *v* = 1608 cm^–1^ (C=N), *v* = 1051 (C–O), *v* = 1454 cm^–1^ (C=C), *v* = 787, 948, 1500 cm^–1^ (Au–C_6_F_5_) MALDI (+) *m*/*z* (%): 1054.066 (89.11) [Au­{C_6_F_5_}_2_Pb–S-terpy]^+^. MALDI (−) *m*/*z* (%): 530.879 (100) [AuC_6_F_5_]^−^. Λ_m_ (methanol): 78 Ω^–1^ cm^2^ mol^–1^.

#### {[{Au­(C_6_F_5_)_2_}_2_{Pb­(S-Terpy)}]·Et_2_O}_2_ (**4**)

A solution of **1** (0.200 g, 1262 mmol) in diethyl ether (10 mL) was stirred for a whole day. Evaporation of the solvent to dryness gave a yellow solid in an almost quantitative yield. ^1^H RMN (400 MHz, [D_6_]-DMSO, 298 K): δ 8.77 [d, 2H, H_1_] [^3^ *J*_H1–H4_ = 4 Hz], 8.66–8.64 [m, 4H, H_2_ + H_3_], 8.04 [pt,2H, H_4_] [(^3^ *J*_H4–H1_) ∼ (^3^ *J*_H4–H7_) = 7.64 Hz], 7.98 [m, 1H, H_5_], 7.82–7.81 [m, 1H, H_9_], 7.54 [m, 2H, H_7_], 7.28 [pt,1H, H_8_] [(^3^ *J*_H8–H9_) ∼ (^3^ *J*_H8–H5_) = 4 Hz], 1.0733 [t,6H, CH_3_] [^3^ *J*_H(CH3)‑H(CH2)_ = 8 Hz]. ^19^F RMN (376.752 MHz, [D_6_]-DMSO, 298 K): δ −162.82 [m, 4F, F_m_], −161.48 [t, 2F, F_p_] [^3^ *J*_p‑m_ = 20 Hz], −114.56 [m, 4F, F_o_]. FT-IR (UATR): *v* = 1599 cm^–1^ (C=N), *v* = 1054 (C–O), *v* = 1451 cm^–1^ (C=C), *v* = 787, 948, 1499 cm^–1^ (Au–C_6_F_5_). MALDI (+) *m*/*z* (%): 1054.066 (64.47) [Au­{C_6_F_5_}_2_Pb–S-terpy]+. MALDI (−) *m*/*z* (%): 530.923 (100) [AuC_6_F_5_]^−^. Elemental analysis calcd (%) for Au_2_PbN_3_SC_47_F_20_H_23_O: C, 34.03; H, 1.40; N, 2.53; S, 1.93. Found C, 34.17; H, 1.34; N, 2.61; S, 1.82. Λ_m_ (methanol): 52 Ω^–1^ cm^2^ mol^–1^.

### Crystallography

Crystals were mounted in inert oil on a MiteGen MicroMountTM and transferred to the cold gas stream of a Bruker APEX-II CCD diffractometer equipped with an Oxford Instruments low-temperature attachment. Data were collected using monochromated Mo Kα radiation (λ = 0.71073 Å). Scan type: ω and ϕ. Absorption corrections: empirical. The structures were solved with the XT structure solution program using Intrinsic Phasing and refined with the ShelXL refinement package using Least Squares minimization and refined on *F* ^2^ using the program SHELXL-2014/7.^29^ One of the C_6_F_5_ rings in **2** is disordered over two different positions (50:50). All non-hydrogen atoms were refined anisotropically. Hydrogen atoms were included using a riding model. Unfortunately, the low quality of the crystals of the diethyl ether derivatives (**3** and **4**) does not yield final crystal data as good as would be desirable, but they allow us to undoubtedly determine the composition and sequence of bonds present in them with accurate enough bond lengths and angles. CCDC 2473062–2473064 contain the supplementary crystallographic data for this paper.

### Computational Details

All calculations were performed using the Gaussian16 suite programs^30^ using the DFT-B3LYP level of theory.^31^ The Karlsruhe def2-TZVP basis sets^32^ were employed for all atoms; 2f-type polarization functions and a pseudorelativistic 60-electron effective core potential (def2- ECP) were used for gold and lead.^33^ TD-DFT calculations were performed to compute the first 50 singlet–singlet excitations and the lowest singlet–triplet excitation.

## Supplementary Material





## Notes

IR spectra, NMR spectra, X-ray data, and optical properties (computational data, population analysis, and molecular orbitals) (PDF)
Cartesian coordinates (XYZ)
