# Supplementary material for: Structural and Photophysical Modulation of Au(I)–Pb(II) Chains by Solvent Inclusion: A Study of Solvatopolymorphs
Source: Organometallics. 2025 Sep 25;44(19):2260–72. doi: 10.1021/acs.organomet.5c00275 (PMC12522680; doi:10.1021/acs.organomet.5c00275)
Supplement: Supplementary file 1 [file om5c00275_si_001.pdf]

Electronic Supplemntary Information (ESI)

Structural and Photophysical Modulation of Au(I)-Pb(II) Chains by Solvent Inclusion: A Study of Solvatopolymorphs

David Royo,[a] Sonia Moreno,[a] María Rodríguez-Castillo,[a] Miguel Monge,[a] M. Elena Olmos,*[a] Fedor Zubkov,[b] Anastasia A. Pronina,[b] Ghodrat Mahmoudi[c,d] and José M. López-de-Luzuriaga*[a]

[a] Departamento de Química, Instituto de Investigación en Química de la Universidad de La Rioja (IQUR), Complejo Científico-Tecnológico, Madre de Dios 53, Universidad de La Rioja, 26006, Logroño, Spain. E-mail: m-elena.olmos@unirioja.es, josemaria.lopez@unirioja.es

[b] Departament of Organic Chemistry, RUDN University, 6 Miklukho-Maklaya St,Moscow 117198. E-mail: fzubkov@sci.pfu.edu.ru , npronina2022@mail.ru

[c] Departamento f Chemistry, Faculty of Science, University of Maragheh, P.O. Box 55136-83111, Maragheh, Iran. E-mail: ghodrathmahmoudi@gmail.com

[d] Samara State Technical University, Molodogvardeyskaya Str 244 Samara 443100 (Russia)

Table of Contents

1. Characterization of the complexes S2

1.1 IR Spectra S2

1.2 ^{1}H NMR SPECTRA (300 MHz, 298K) S4

1.3 ^{19}F NMR spectrum (300 MHz, 298K) S7

2. Syngle crystal analysis of compounds 1-3 .... S9

3. Powder X-ray diffraction analysis S10

4. Optical properties S12

1. Characterization of the complexes

1.1 IR Spectra

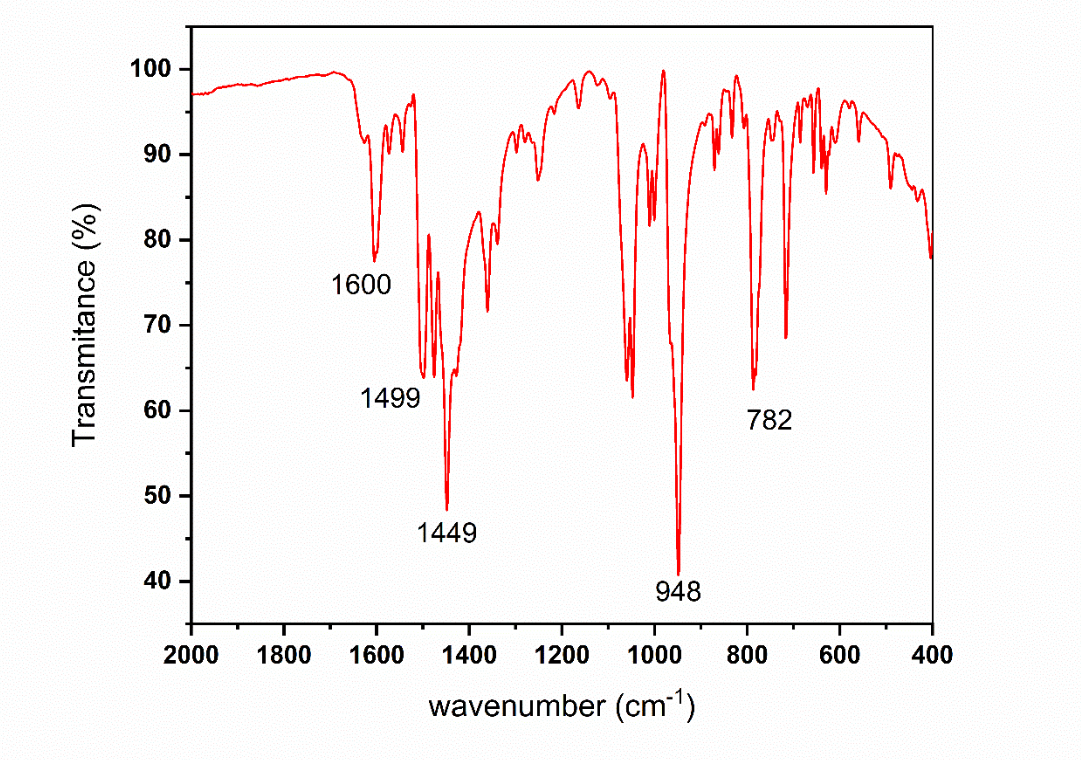

Figure S1: FT-IR spectrum of complex **1**.

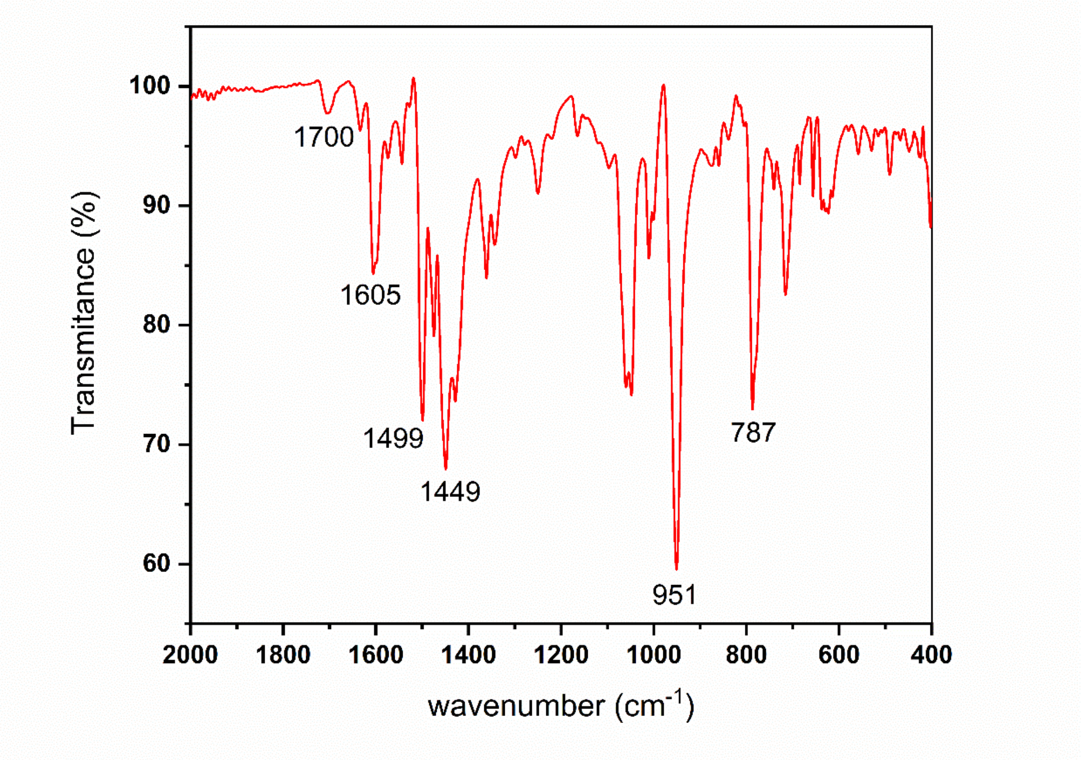

Figure S2: FT-IR spectrum of complex **2**.

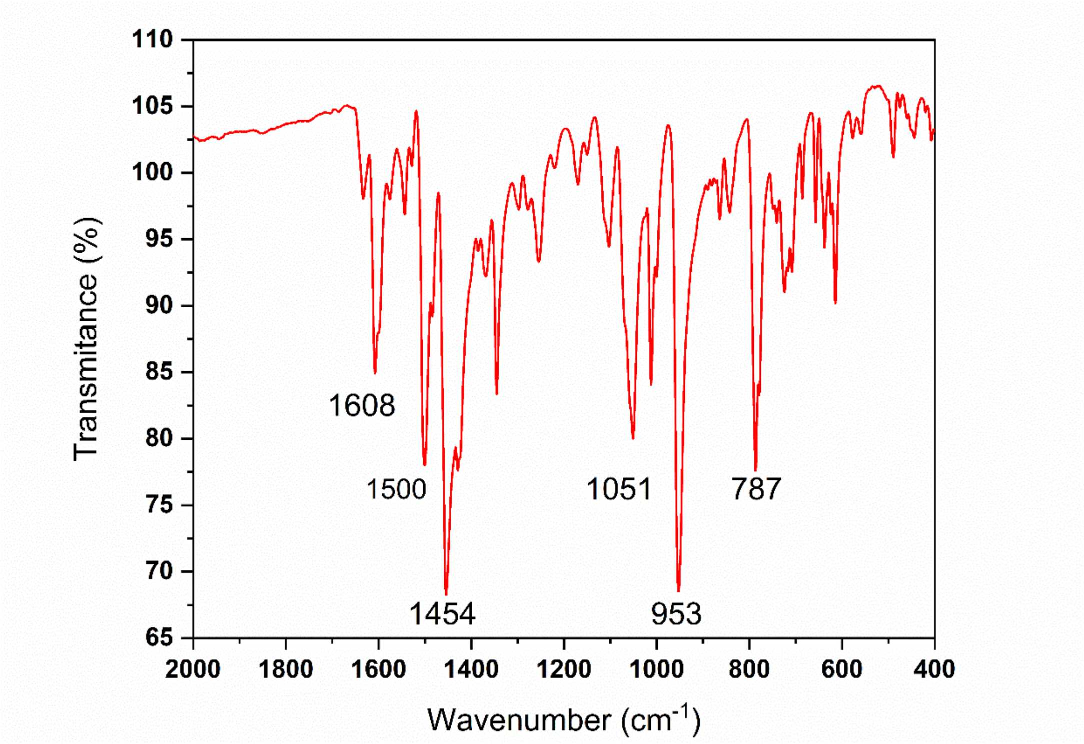

Figure S3: FT-IR spectrum of complex **3**.

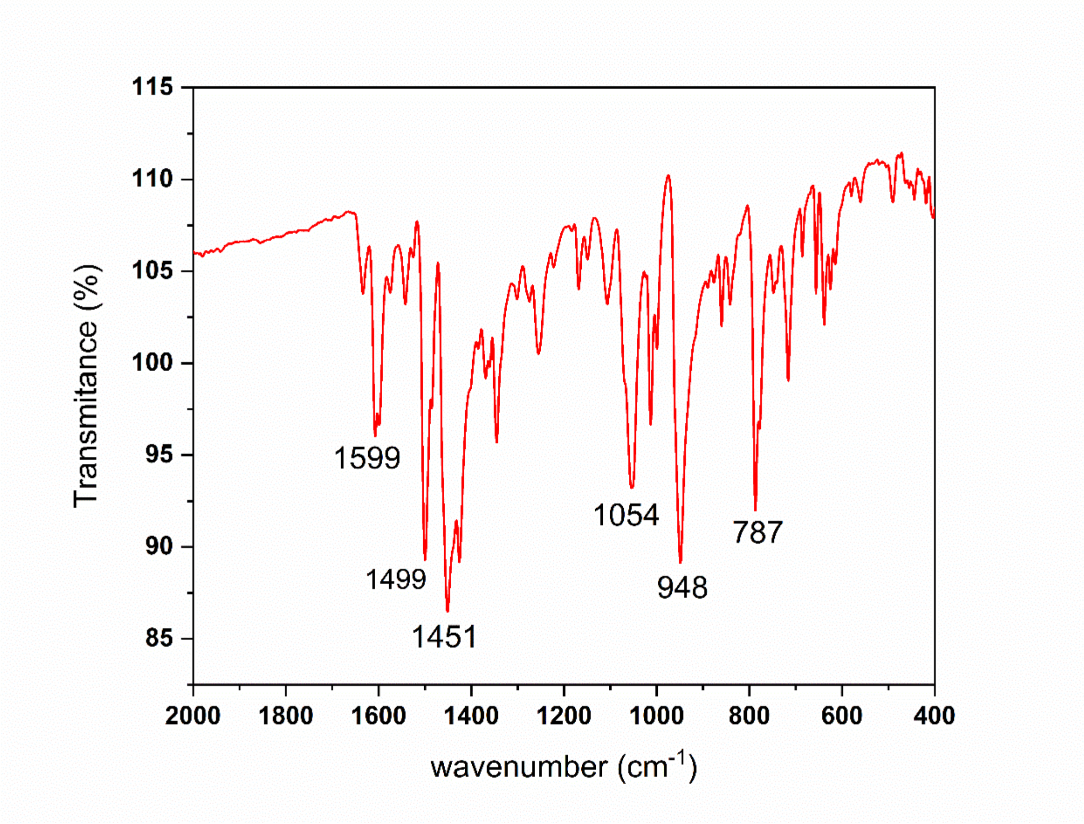

Figure S4: FT-IR spectrum of complex **4**.

1.2 ^{1}H NMR SPECTRA (300 MHz, 298K)

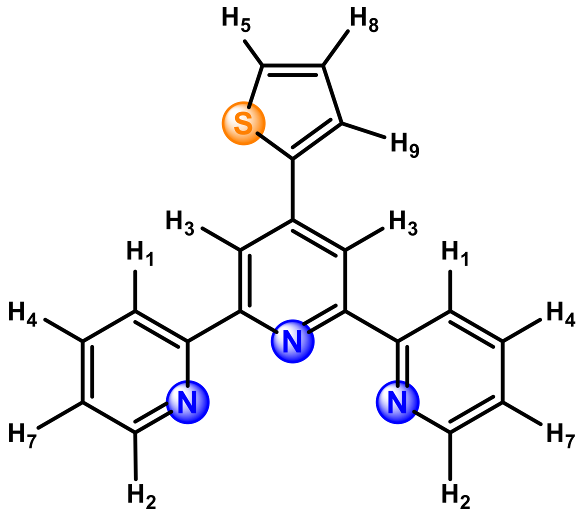

3

Figure S5: ^{1}H NMR assignment of ligand S-Terpy.

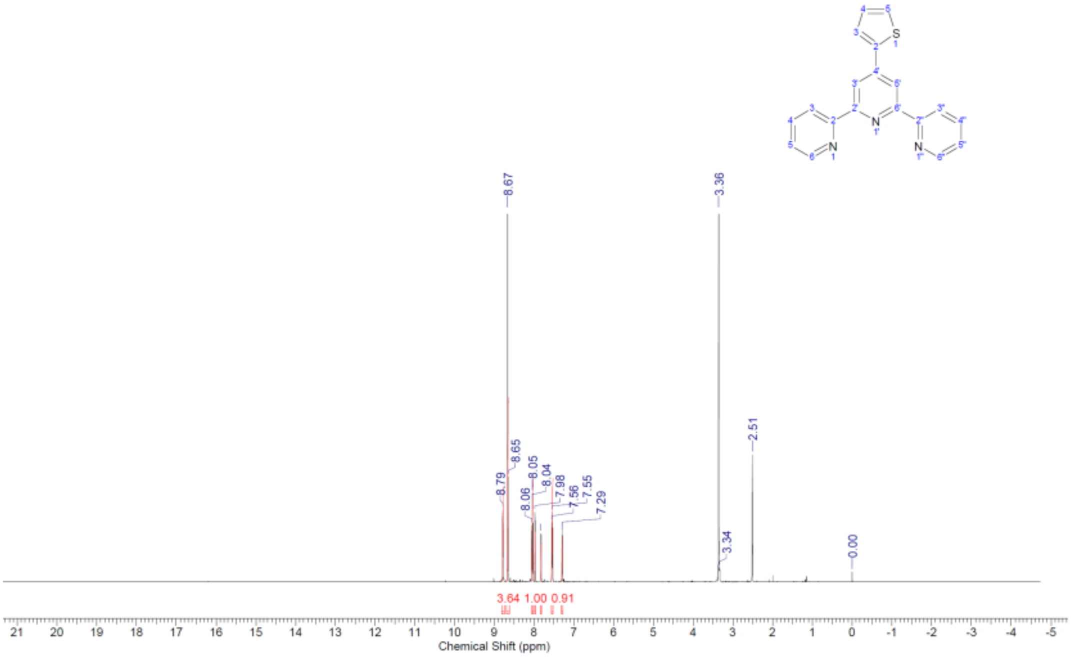

Figure S6: ^{1}H NMR spectrum of S-Terpy in [D_6]-DMSO ligand S-Terpy.

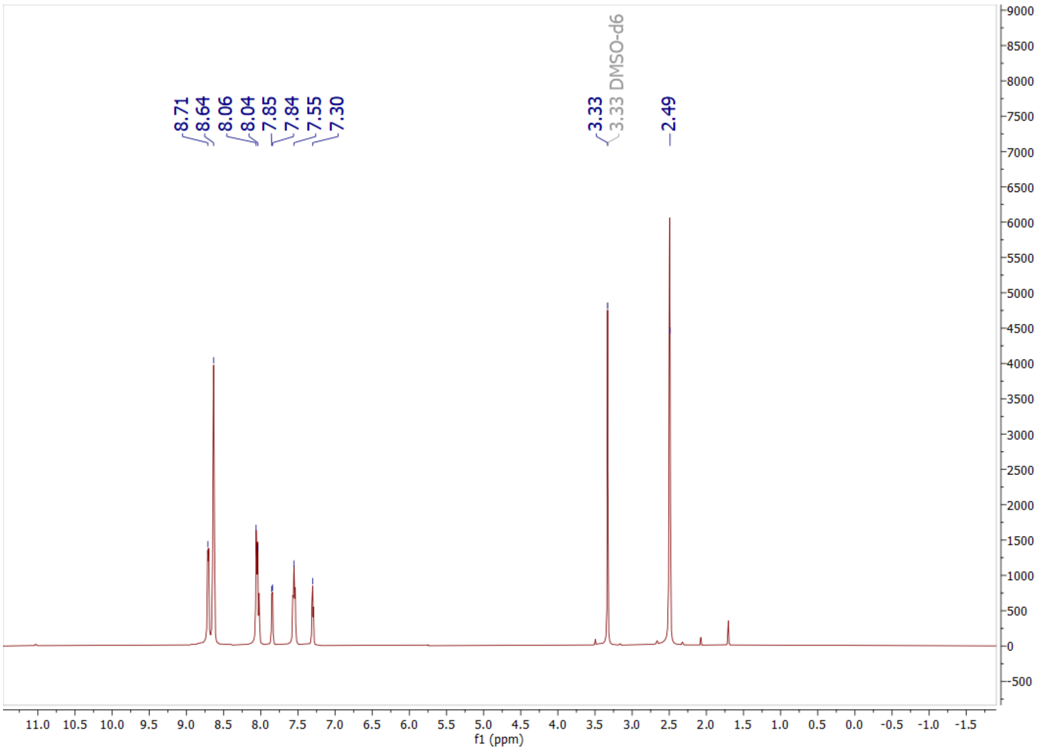

Figure S7: ^{1}H NMR spectrum of complex **1** in [D$_6$]-DMSO.

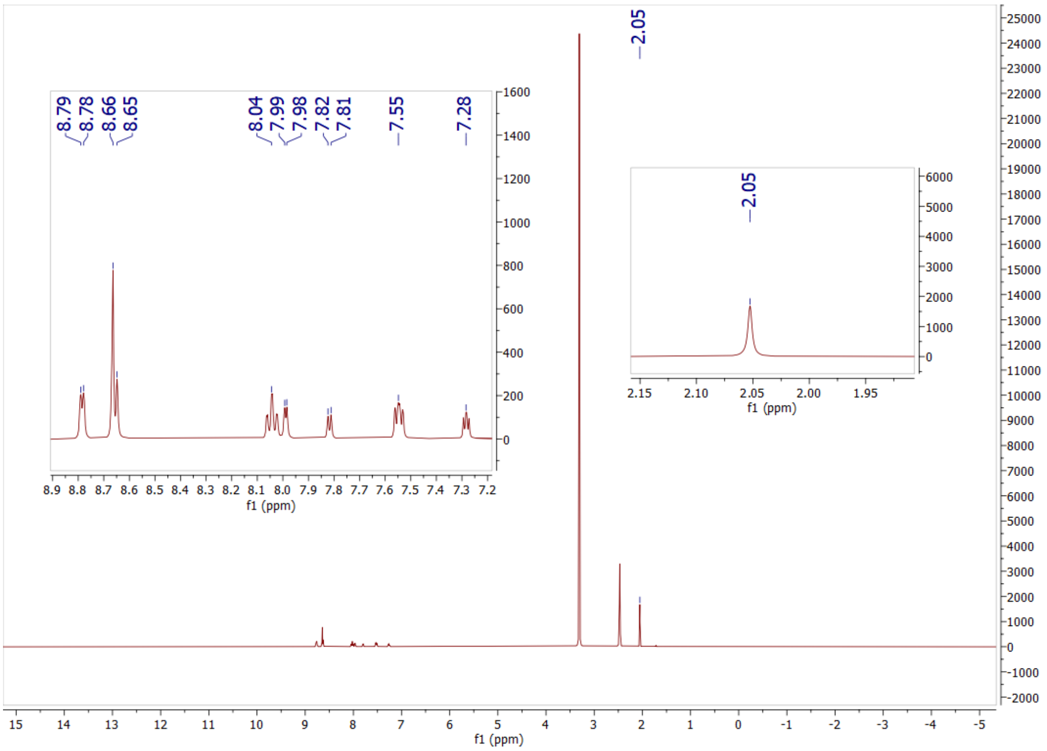

Figure S8: ^{1}H NMR spectrum of complex **2** in [D$_6$]-DMSO.

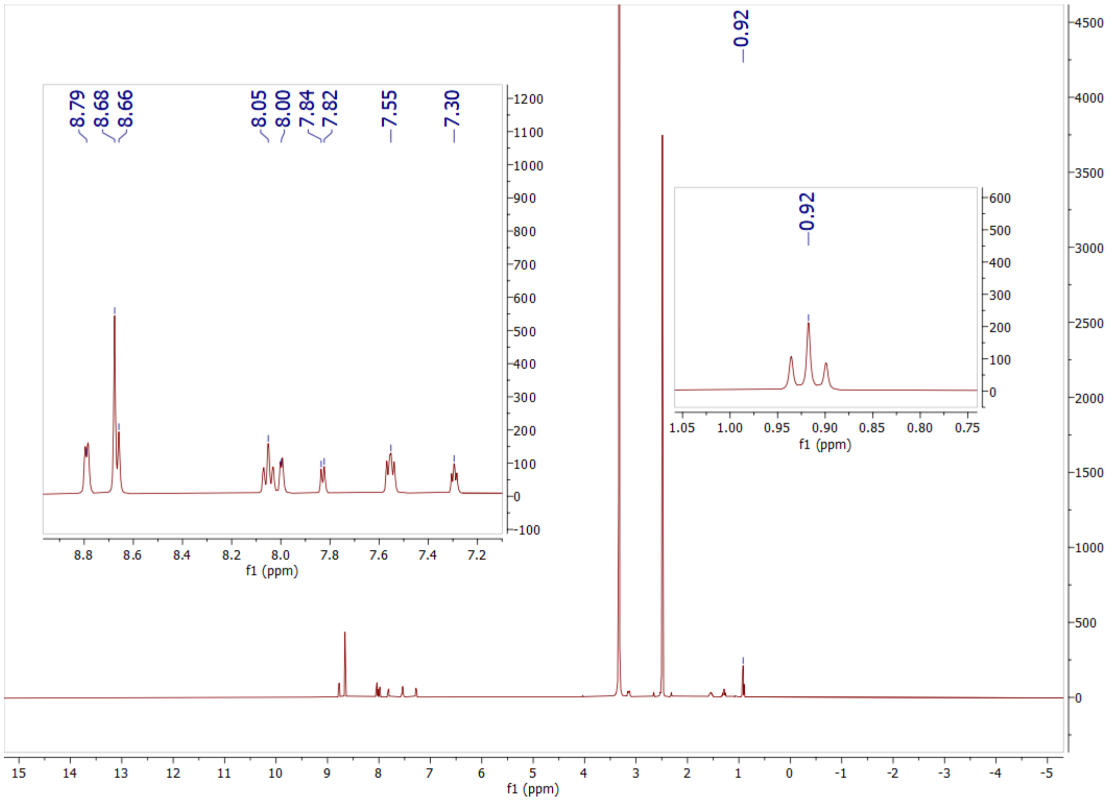

Figure S9: ^{1}H NMR spectrum of complex **3** in $[D_6]$-DMSO.

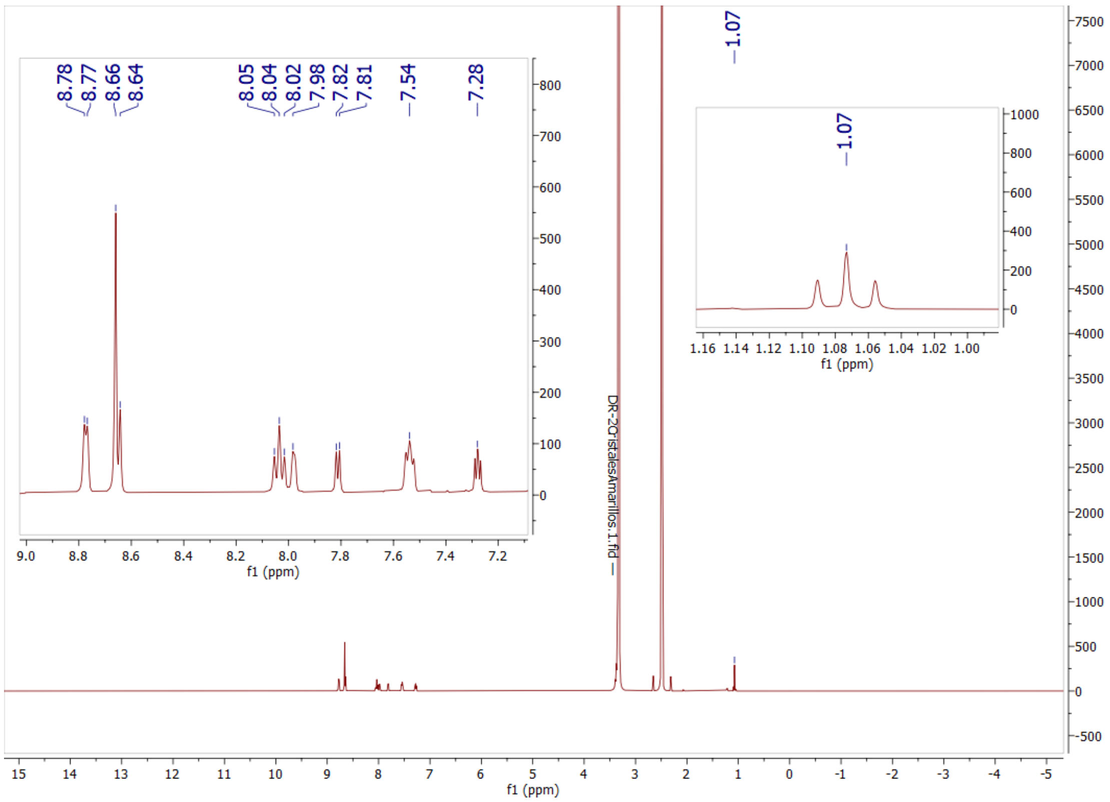

Figure S10: ^{1}H NMR spectrum of complex **4** in $[D_6]$-DMSO

1.3 ^{19}F NMR spectrum (300 MHz, 298K)

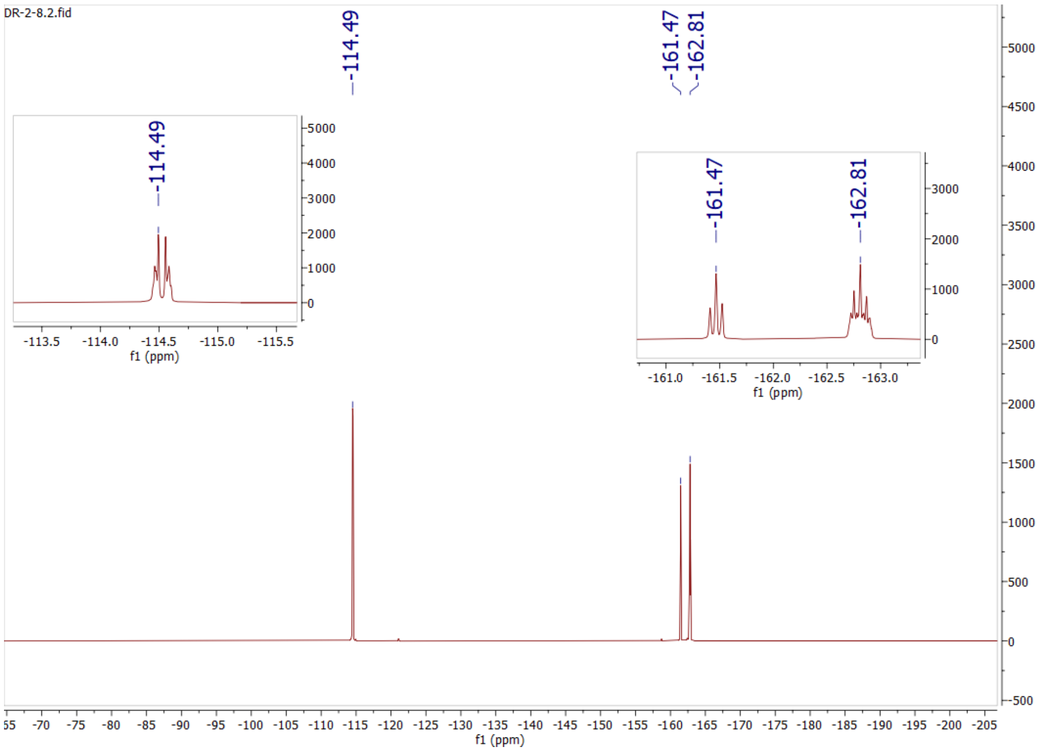

Figure S11: ^{19}F NMR spectrum of complex **1** in [D$_6$]-DMSO.

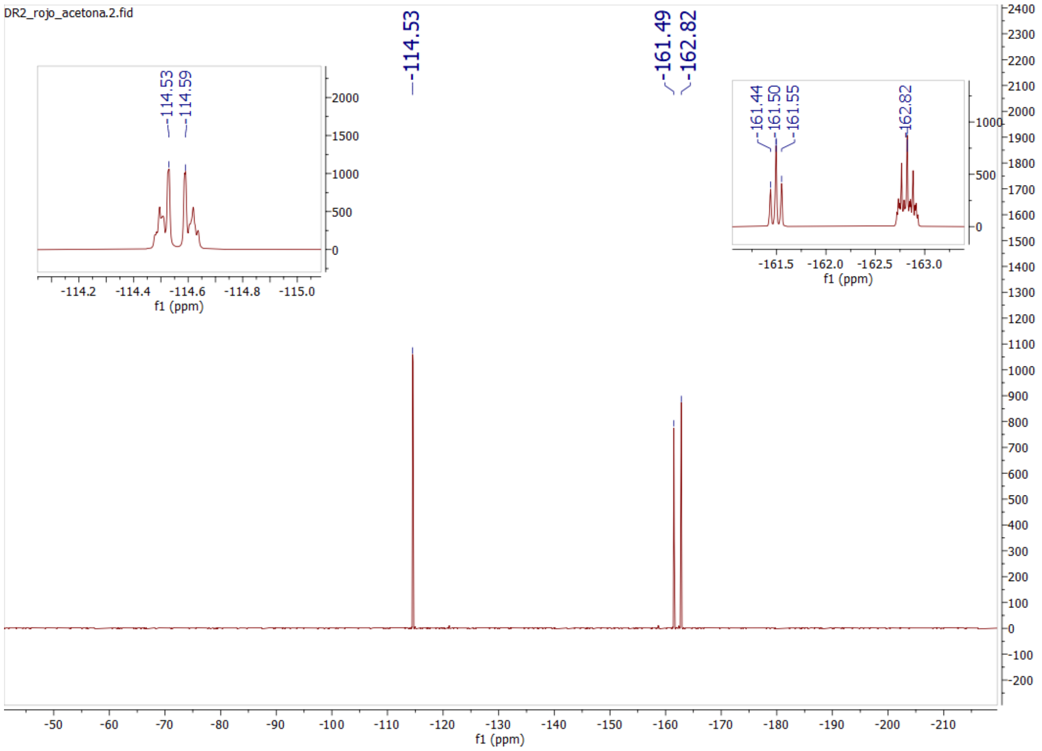

Figure S12: ^{19}F NMR spectrum of complex **2** in [D$_6$]-DMSO.

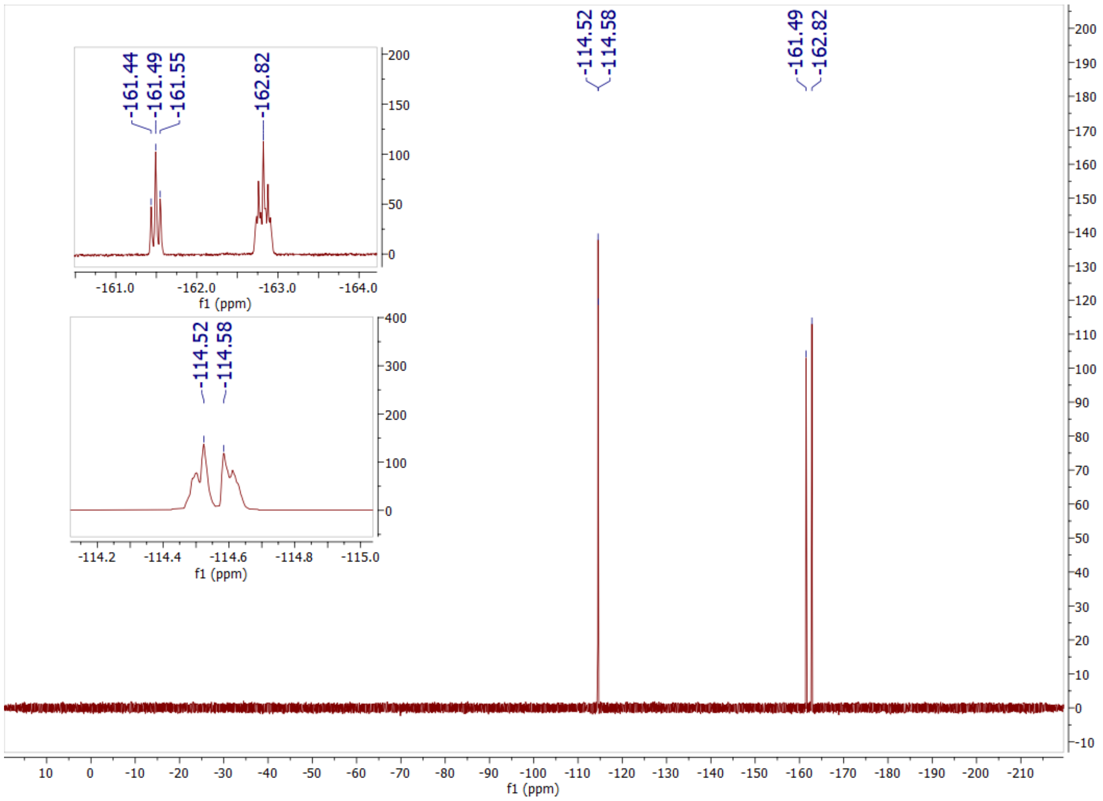

Figure S13: ^{19}F NMR spectrum of complex **3** in [D_6]-DMSO.

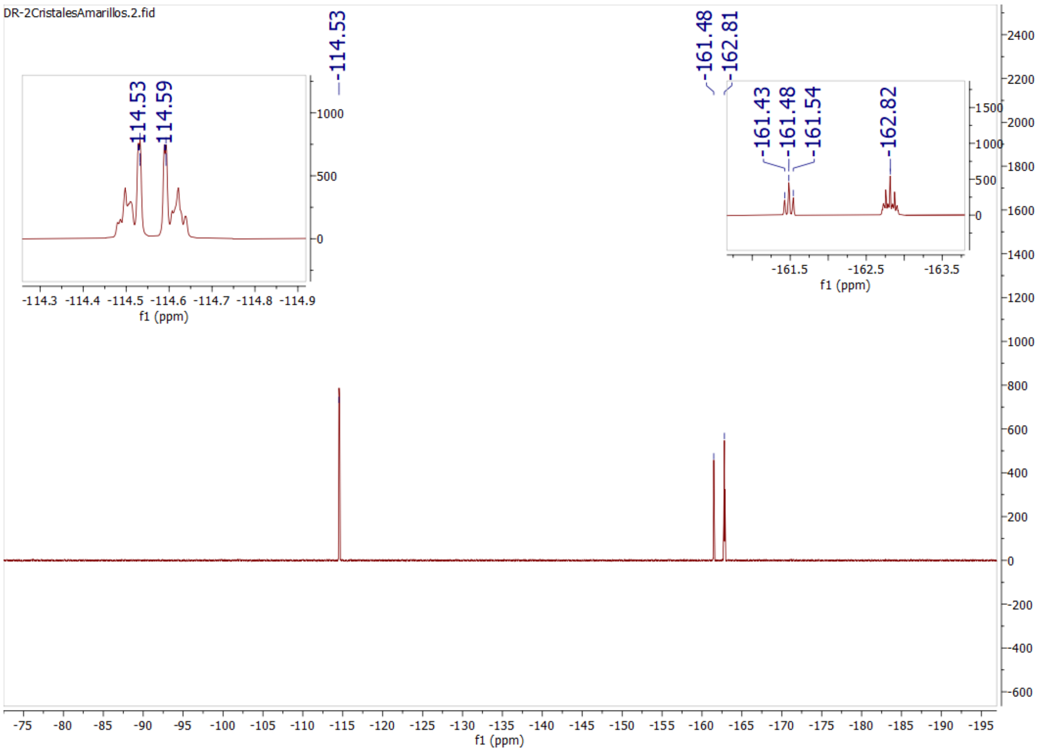

Figure S14: ^{19}F NMR spectrum of complex **4** in [D_6]-DMSO.

2. Syngle crystal analysis of compounds 1-3

	**2**	**3**	**4**
Chemical Formula	$C_{43}H_{13}Au_2F_{20}N_3PbS·0.5C_3H_6O$	$C_{43}H_{13}Au_2F_{20}N_3PbS$ · 0.5 $C_4H_{10}O$	$C_{43}H_{13}Au_2F_{20}N_3PbS$ · $C_4H_{10}O$
Crystal habit	Dark-green	Green-yellow	red
Crystal size /mm	0.321 x 0.060 x 0.018	0.481 x 0.116 x 0.077	0.225 x 0.132 x 0.026
Crystal system	Monoclinic	Triclinic	Triclinic
Space group	P 2_1/c	P -1	P -1
a/Å	19.0805(13)	16.773(18)	13.4159(10)
b/Å	13.0614(8)	17.31(2)	13.7485(13)
c/Å	17.9017(12)	19.58(3)	15.0120(13)
α/°	90	64.44(4)	68.617(3)
β/°	106.869(3)	71.99(4)	83.194(3)
γ/°	90	80.33(4)	72.859(3)
V/Å^3	4269.5(5)	4874(10)	2463.6(4)
Z	4	4	2
D_c/g cm^{-3}	2.5111	2.210	2.236
M	1613.79	1621.81	1658.87
F(000)	2976	2996	1540
T/°C	100 (2) K	298(2)k	100(2)K
2θmax/°	56	48	49
μ(Mo-Kα)/mm^{-1}	10.967	9.607	9.507
No. refl. Measured	10144	20900	83343
No. unique refl.	10144	15258	8375
R_{int}	0.0597	0.0618	0.1654
R[F>2σ(F)][a]	0.0610	0.1036	0.0902
wR[F 2 , all refl.][b]	0.1268	0.3069	0.2736
No. of refl. Used [F>2σ(F)]	10144	15258	8375
No. of parameters	705	767	606
No. of restrains	209	108	10
S [c]	1.286	0.973	1.105
Max. residual electron density/e·Å^{-3}	2.105	2.323	5.656

3. Powder X-ray diffraction analysis

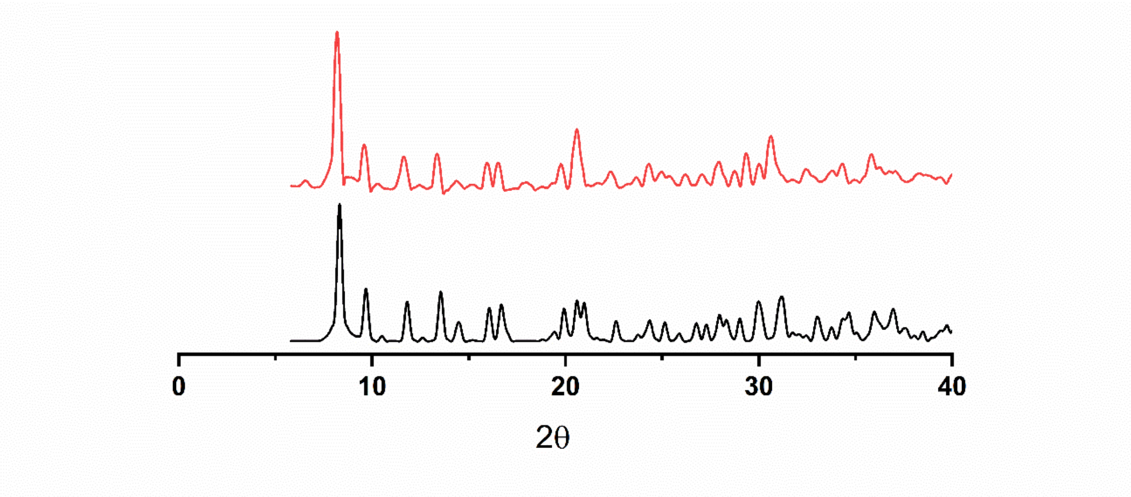

Figure S15: Theoretical (black) and experimental (red) X-ray powder pattern of complex **2**.

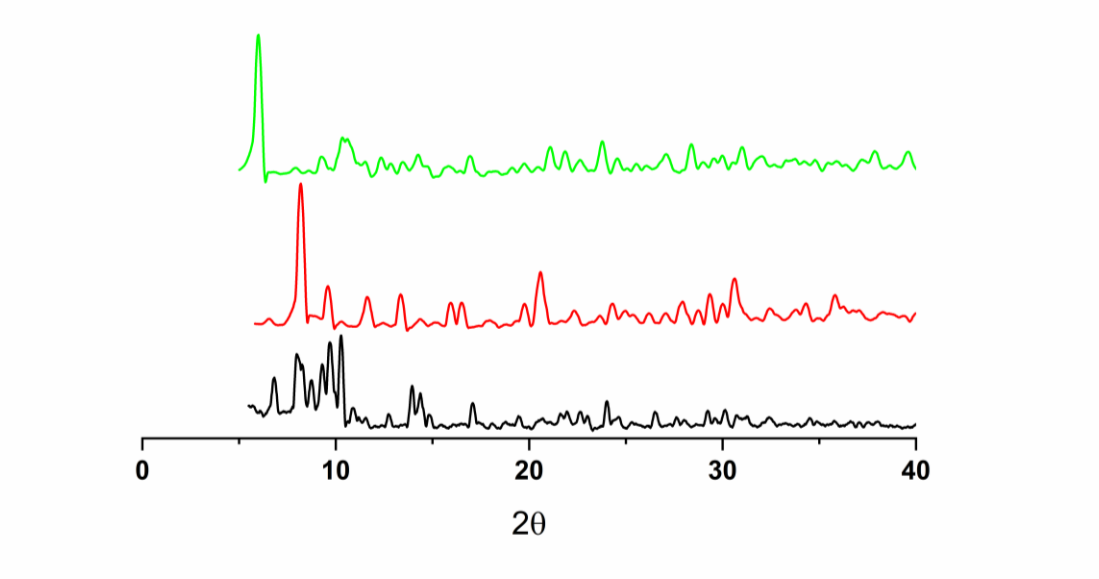

Figure S16: X-ray diffraction spectra of powder obtained by treatment of compound **1** (black) with acetone (compound **2** red) and diethyleter (compound **4** green)

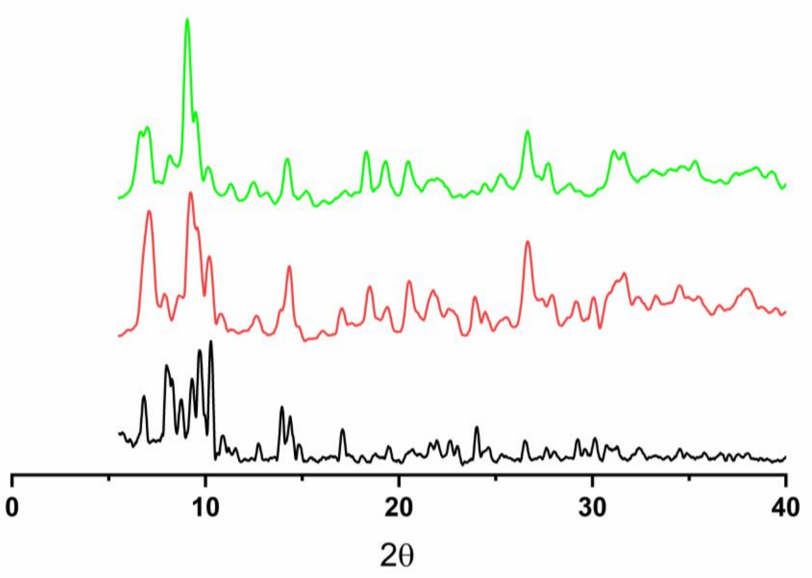

Figure S17: X-ray diffraction spectra of compound **1** (black) compound **2** after heat application (red) and compound **4** after heat application (green).

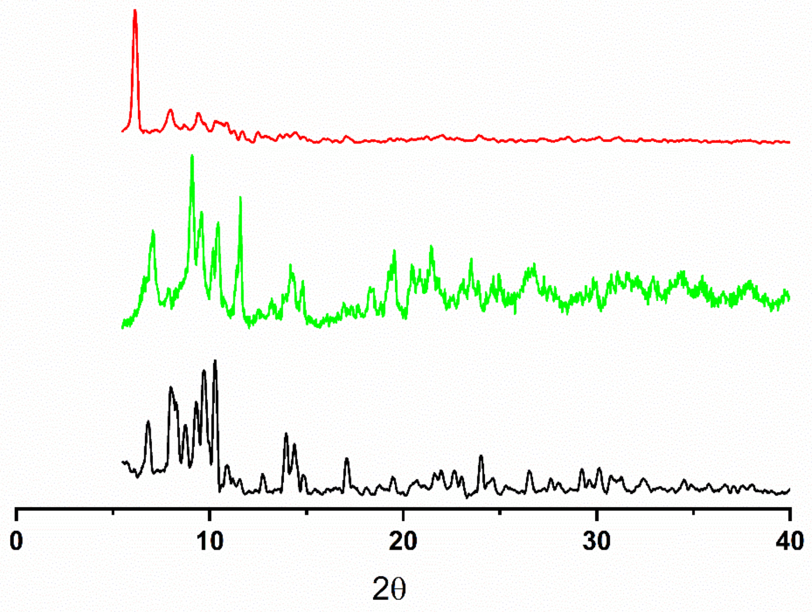

Figure S18: X-ray diffraction spectra of compound **1** (black) compound **2** after pressure application (green) and compound **4** after pressure application (red).

4. Optical properties

Table S1: Selected frontier molecular orbital composition (%) for model **1**.

Complex	Orbital	Au	Pb	C_6F_5	S-Terpy
1	LUMO+3	2	3	3	92
	LUMO+1	13	19	10	58
	LUMO	2	-	2	95
	HOMO	-	-	2	95
	HOMO-1	64	11	21	4
	HOMO-3	14	-	84	2
	HOMO-5	16	2	80	3
	HOMO-6	2	-	96	1
	HOMO-7	4	-	88	4
	HOMO-10	2	-	93	4

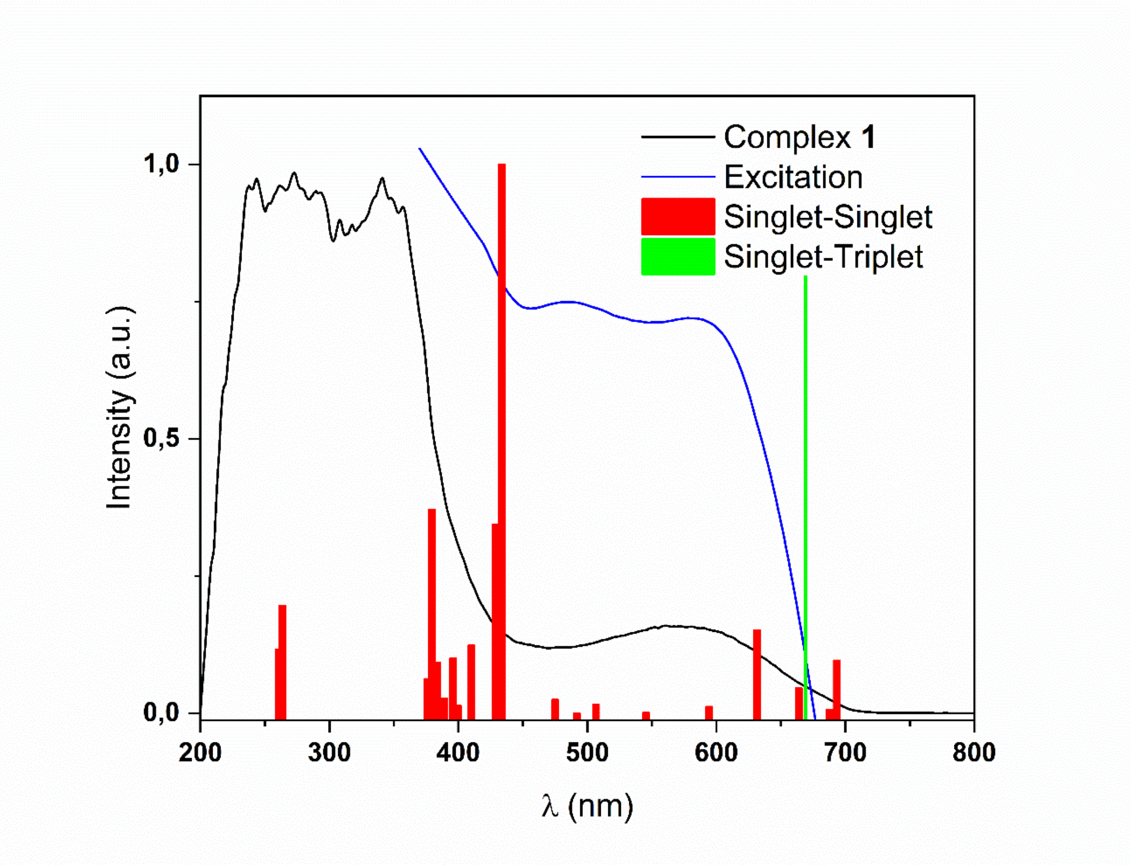

Figure S19: Experimental UV-vis solid state absoption spectrum (black line), excitation (blue) TD-DFT singlet-singlet excitations (red bars) and singlet-triplet (green bars) fo complex **1**. The green bar only represents the energy of the lowest singlet-triplet transitions since the oscillator strength cannot be calculated.

Table S2: Selected frontier molecular orbital composition (%) for complex **2**.

Complex	Orbital	Au	Pb	C_6F_5	S-Terpy	Dvte
2	LUMO+1	3	8	4	85	0
	LUMO	6	9	6	79	0
	HOMO	64	9	24	3	0
	HOMO-7	6	0	82	7	3
	HOMO-8	5	1	59	34	1
	HOMO-14	9	0	82	7	0

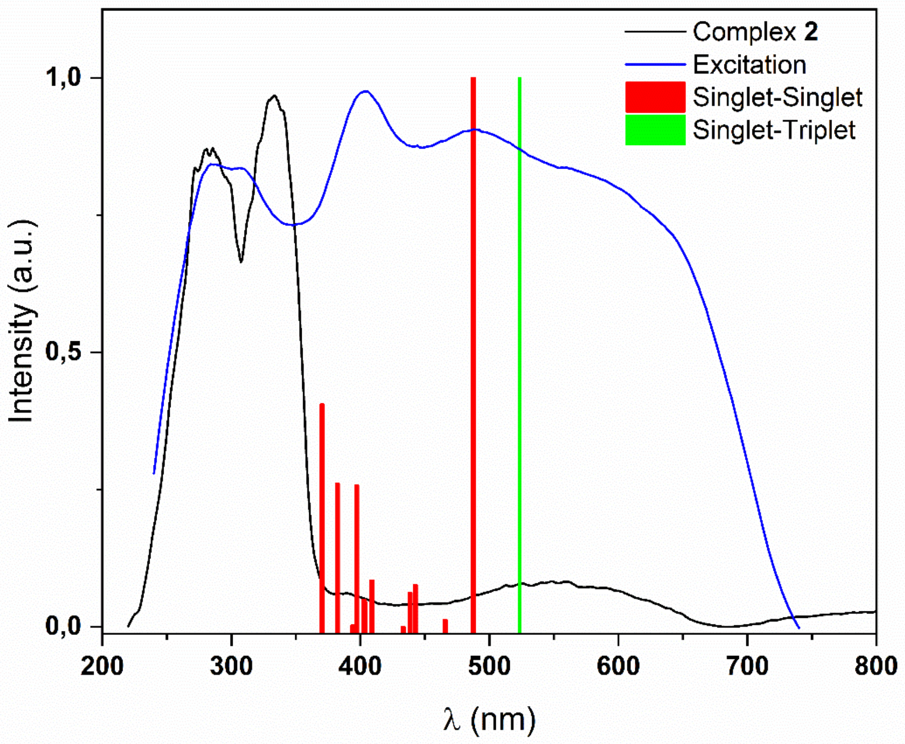

Figure S20: Experimental UV-vis solid state absoption spectrum (black line), excitation (blue) TD-DFT singlet-singlet excitations (red bars) and singlet-triplet (green bars) for complex **2**. The green bar only represents the energy of the lowest singlet-triplet transitions since the oscillator strength cannot be calculated.

Table S3: Selected frontier molecular orbital composition (%) for complex **3**.

Complex 3	Orbital	Au	Pb	S-Terpy	C_6F_5	Dvte
**3**	LUMO+1	4	6	86	5	0
	LUMO	7	13	70	9	0
	HOMO	63	12	4	19	0
	HOMO-4	0	0	2	98	0
	HOMO-6	3	0	36	61	0
	HOMO-9	2	0	37	57	4
	HOMO-11	5	0	15	79	0
	HOMO-14	21	5	9	36	29

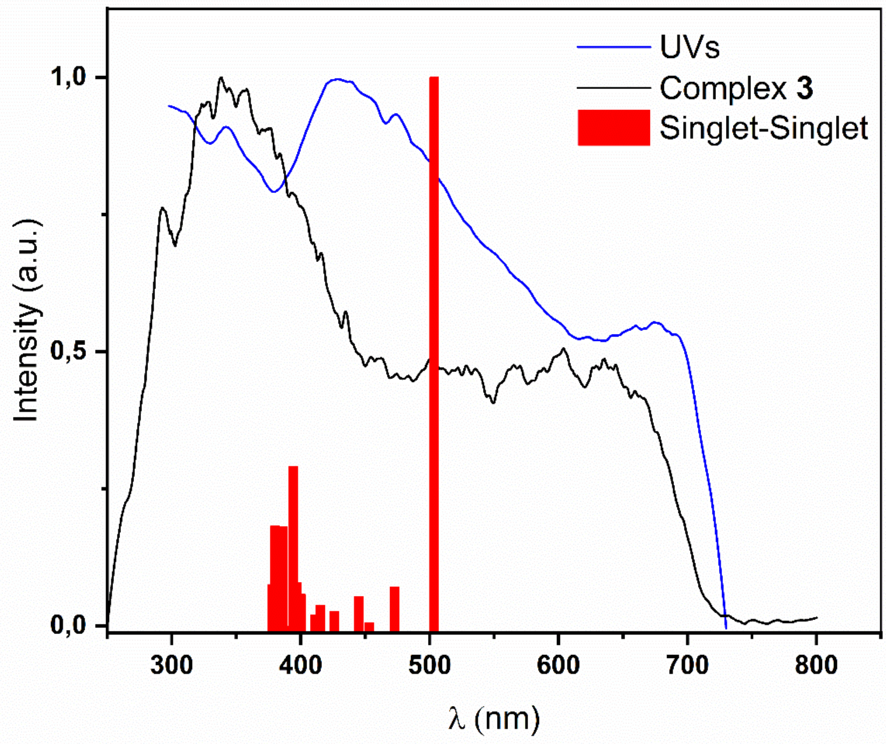

Figure S21: Experimental UV-vis solid state absoption spectrum (black line), excitation (blue) TD-DFT singlet-singlet excitations (red bars) for complex **3**.

Table S4: Selected frontier molecular orbital composition (%) for complex **4**.

Complex 4	Orbital	Au	Pb	S-Terpy	C6F5
4	LUMO+5	11	18	56	15
	LUMO+4	20	26	38	16
	LUMO+1	11	5	84	5
	LUMO	8	11	74	5
	HOMO	69	11	2	19
	HOMO-2	12	0	10	77
	HOMO-4	6	0	40	55
	HOMO-5	1	0	38	60
	HOMO-7	3	0	18	77
	HOMO-9	2	0	13	82
	HOMO-14	13	2	25	59

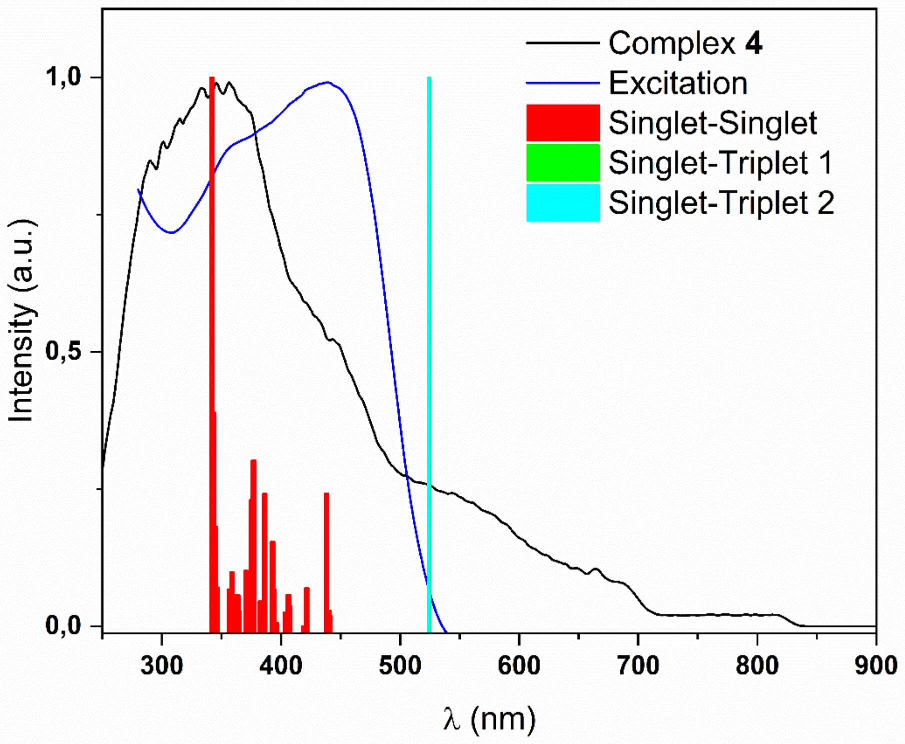

Figure S22: Experimental UV-vis solid state absoption spectrum (black line), excitation (blue) TD-DFT singlet-singlet excitations (red bars) and singlet-triplet (green bars) for complex **4**. The green bar only represents the energy of the lowest singlet-triplet transitions since the oscillator strength cannot be calculated.

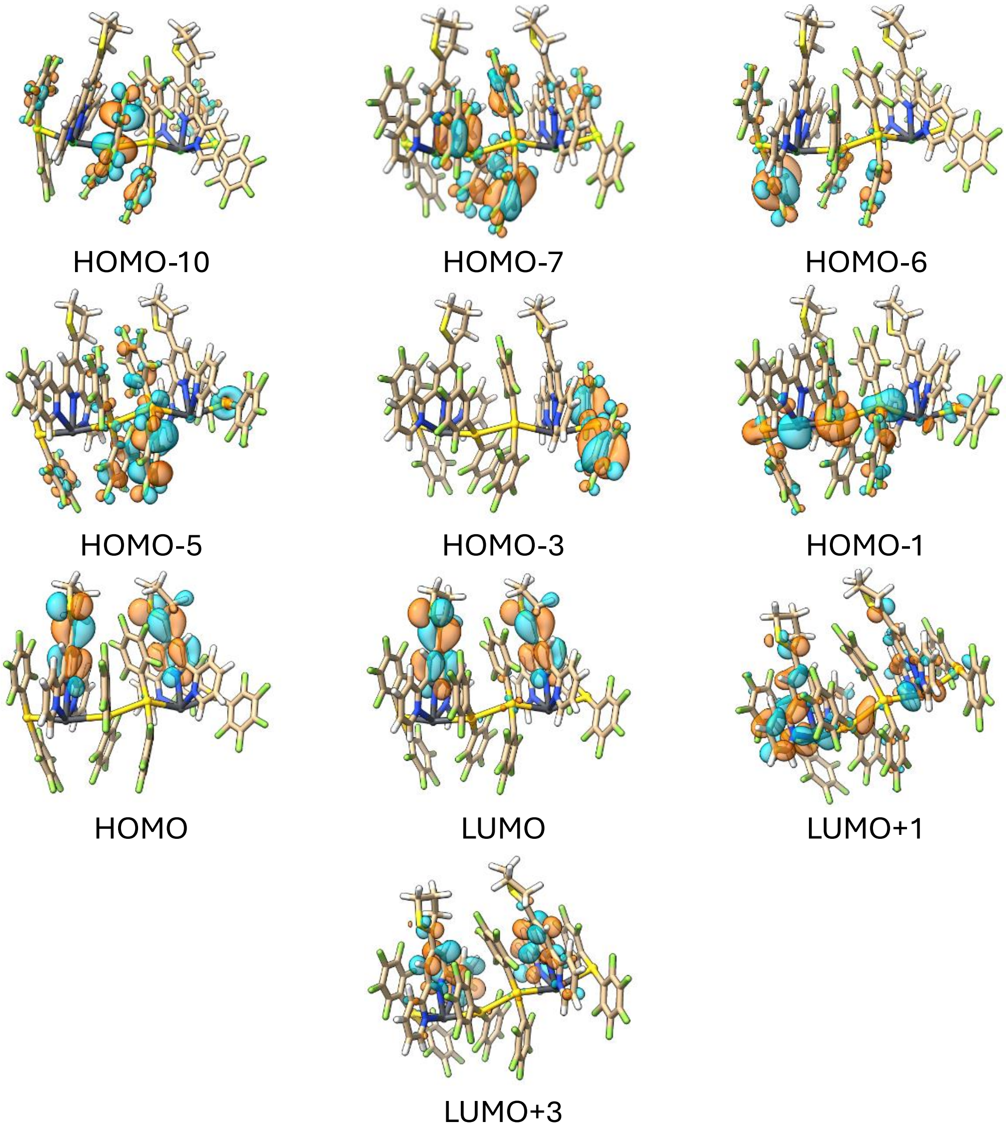

Figure S23: Molecular orbitals involved in the most important transitions calculated for complex **1**.

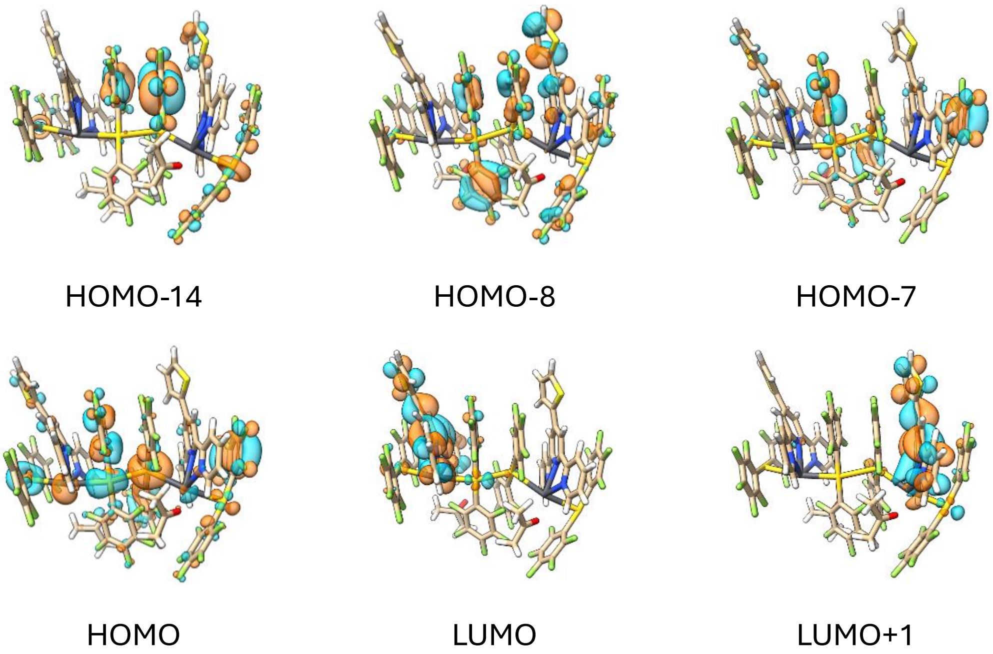

Figure S24: Molecular orbitals involved in the most important transitions calculated for complex **2**.

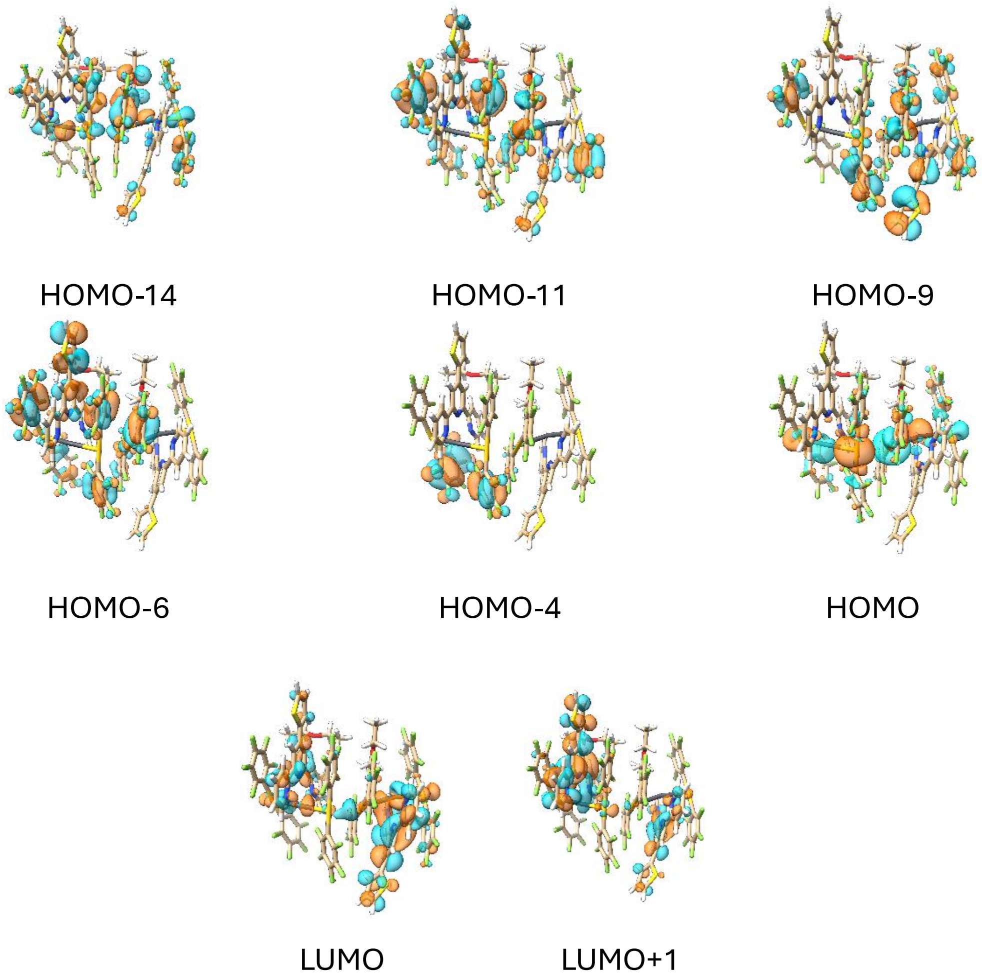

Figure S25: Molecular orbitals involved in the most important transitions calculated for complex **3**.

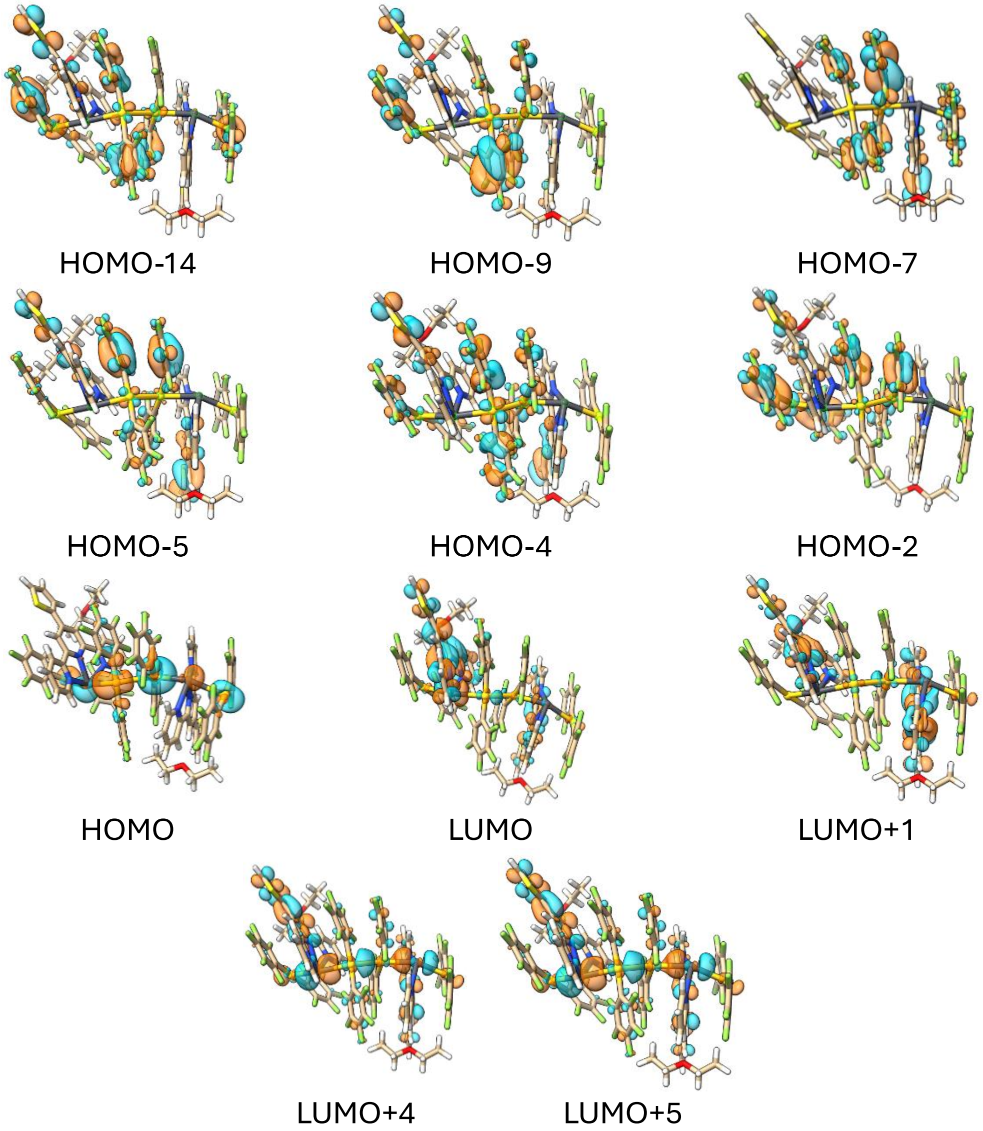

Figure S26: Molecular orbitals involved in the most important transitions calculated for complex **4**.
